# Cleaving DNA with DNA: Cooperative Tuning of Structure and Reactivity Driven by Copper Ions

**DOI:** 10.1002/advs.202306710

**Published:** 2024-02-28

**Authors:** Sarath Chandra Dantu, Mahdi Khalil, Marc Bria, Christine Saint‐Pierre, Maylis Orio, Didier Gasparutto, Giuseppe Sicoli

**Affiliations:** ^1^ Department of Computer Science Brunel University London Kingston Lane Uxbridge UB8 3PH UK; ^2^ LASIRE CNRS UMR 8516 University of Lille C4 building, Avenue Paul Langevin Villeneuve d'Ascq F‐59655 France; ^3^ Michle‐Eugène Chevreul Institute FR 2638, Avenue Paul Langevin Villeneuve d'Ascq F‐59655 France; ^4^ Université Grenoble Alpes CEA CNRS UMR 5819 SyMMES‐CREAB, Avenue des Martyrs Grenoble F‐38000 France; ^5^ Aix Marseille Université CNRS Centrale Marseille iSm2, UMR CNRS 7313 Marseille 13397 France

**Keywords:** DNAzymes, hyperfine spectroscopy, metal soup, multiple binding, radical path

## Abstract

A copper‐dependent self‐cleaving DNA (DNAzyme or deoyxyribozyme) previously isolated by in vitro selection has been analyzed by a combination of Molecular Dynamics (MD) simulations and advanced Electron Paramagnetic Resonance (Electron Spin Resonance) EPR/ESR spectroscopy, providing insights on the structural and mechanistic features of the cleavage reaction. The modeled 46‐nucleotide deoxyribozyme in MD simulations forms duplex and triplex sub‐structures that flank a highly conserved catalytic core. The DNA self‐cleaving construct can also form a bimolecular complex that has a distinct substrate and enzyme domains. The highly dynamic structure combined with an oxidative site‐specific cleavage of the substrate are two key‐aspects to elucidate. By combining EPR/ESR spectroscopy with selectively isotopically labeled nucleotides it has been possible to overcome the major drawback related to the “metal‐soup” scenario, also known as “super‐stoichiometric” ratios of cofactors versus substrate, conventionally required for the DNA cleavage reaction within those nucleic acids‐based enzymes. The focus on the endogenous paramagnetic center (Cu^2+^) here described paves the way for analysis on mixtures where several different cofactors are involved. Furthermore, the insertion of cleavage reaction within more complex architectures is now a realistic perspective towards the applicability of EPR/ESR spectroscopic studies.

## Graphical abstract

Within the DNA‐cleaving‐DNA process, copper ions exhibit a “multitasking” role, ranging from stabilizing 3D architectures to production of reactive oxygen species (ROS), mandatory for the DNA cleavage. EPR spectroscopy has disentangled different functions of the metal cofactor(s) both for coordination sphere and mechanistic insights.

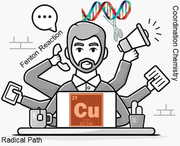

## 1 Introduction

DNA cleavage is a vital process in all living systems. Topoisomerase enzymes resolve topological problems of DNA in replication, transcription and other cellular transactions by cleaving one or both strands of the DNA.^[^ ^1^ ^]^ Restriction enzymes protect the cell against virus infection by cleavage of the foreign DNA^[^ ^2^ ^]^ or by degrading cellular DNA during apoptosis of the affected cell.^[^ ^3^ ^]^ The activity of many anticancer drugs rely on their ability to introduce extended damage to the DNA in the (affected) cells (e.g., bleomycin),^[^ ^4^ ^]^ which can trigger apoptosis,^[^ ^5^ ^]^ leading to the cell death.^[^ ^6^ ^]^

DNA molecules, as RNA and proteins, are capable of folding into well‐defined three‐dimensional structures that can catalyze chemical reactions. Since the selection of the first DNAzyme,^[^ ^7^ ^]^ the number of DNA catalysts has dramatically increased and their catalytic repertoire has exceeded the cleavage of RNA.^[^ ^8^ ^]^ In addition, DNA‐catalyzed reactions involving oligonucleotide substrates are DNA ligation,^[^ ^9^ ^]^ DNA cleavage,^[^ ^10^ ^]^ site‐specific thymidine excision, DNA phosphodiester hydrolysis,^[^ ^11^ ^]^ DNA phosphorylation,^[^ ^12^ ^]^ DNA adenylylation/capping,^[^ ^13^ ^]^ and RNA ligation.^[^ ^14^ ^]^

**Scheme 1:**
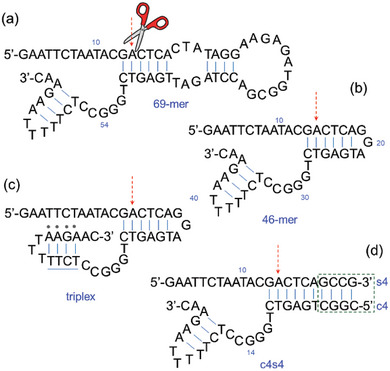
Secondary structures of the DNAzymes (deoxyribozymes) cleaving DNA: a) the first DNA‐cleaving‐DNA formed by 69 nucleotides has been followed by the b) 46mer as reduced structure containing the catalytic core. c) for the mono‐molecular 46‐mer a triplex version can be obtained. d) The bimolecular structure (c4s4) formed by substrate and enzyme oligomers, both containing four additional nucleotides (GCCG / CGGC base paring; c4s4 nomenclature derives from those four bases both on the catalyst and on the substrate replacing the GGA bulge fragment, to stabilize the secondary structure).

A self‐cleaving domain that was obtained through in vitro selection has been a 69mer (69 nucleotides in length, **Scheme** **1**a).^[^ ^15^ ^]^ The predicted secondary structure for this class of deoxyribozymes includes two stem‐loops that are interspersed with three single‐stranded domains. In addition, a contiguous series of 21 nucleotides comprising the 3′ terminus of variant self‐cleaving DNAs remained highly conserved throughout the in vitro selection process, indicating that these nucleotides are critical for deoxyribozyme function.^[^ ^15^ ^]^ Replacement of the 26 nucleotides from the 69mer DNA with a “tri‐loop”, enhances the stability of adjoining stem structures; the shorter self‐cleaving DNA (46mer) retains this conserved sequence domain (Scheme 1b) as well as the Cu^2+^‐dependent self‐cleavage at the same catalytic rate as the original full‐length deoxyribozyme.^[^ ^16^ ^]^

A triplex architecture can be achieved for the 46mer, and it can be also divided into separate substrate and catalyst domains that are represented (Scheme 1c,d). Indeed, oligonucleotides s4 (substrate) and c4 (catalyst) can be obtained. The substrate DNA includes the primary site of DNA cleavage, while the catalyst DNA contains the original random‐sequence domain that also contains all nucleotides that were conserved during the in vitro selection process. When mixed, these DNAs form a bimolecular complex that retains full catalytic activity. Oxidation at the C1′, C4′, or C5′ positions of deoxyribose are putative sites for hosting the cleavage process.^[^ ^17^ ^]^ Random cleavage of DNA is readily observable upon incubation of polynucleotides in the presence of millimolar concentrations of mono‐ or divalent copper when combined with a similar concentration of ascorbate ^[^ ^18^ ^]^ Several DNA‐cleaving agents such as 1,10‐phenanthroline dramatically enhance the rate of DNA cleavage by Cu^2+^ and ascorbate, even when both Cu^2+^ and the DNA‐cleaving agent are used at micromolar concentrations.^[^ ^19^ ^]^ Supplementing these oxidative cleavage reactions with H_2_O_2_ produces even greater rates of DNA chain cleavage, while enzymatically removing H_2_O_2_ from the reaction using catalase prevents the cleavage of DNA.^[^ ^20^ ^]^

Several significant drawbacks hinder the use of class II DNAs as artificial restriction enzymes for single‐stranded DNA. First, the substrate strand contains nucleotides whose base identities are critical for catalytic activity. Therefore, the range of DNA sequences that can be cleaved is restricted thereby precluding the targeted cleavage of any DNA sequence. Second, the catalyst strand can also be cleaved during the reaction. Third, oxidative DNA cleavage yields some products that cannot easily be used in other molecular biology protocols due to the nucleoside fragments that are retained by some phosphate groups. Fourth, oxidative cleavage results in the loss of sequence information, as a base is destroyed during DNA strand scission. It is worth to note that a common denominator for the mentioned cleavage reactions is the “super‐stoichiometric ratio” (also known as “non‐stoichiometric ratio”, or “metal‐soup” or “bath of electron spins”) used for the cofactor (i.e., Cu^2+^) which represents a deterrent for several conventional spectroscopic techniques. With respect to DNA cleavage, RNA ligation catalyzed by DNAzyme may count on recent computational and crystal structures studies,^[^ ^21^, ^22^, ^23^ ^]^ but these studies provide information exclusively on the “post‐catalytic” structure (product(s) of reaction) and not the pre‐catalytic or catalytic complex. To date, insights on mechanistic and structural features related to “DNAs‐cleaved by DNAzymes”, are severely lacking. A recent study on RNA‐cleaving DNA catalyst support the structural plasticity of the 10–23 DNAzymes architecture;^[^ ^8^ ^]^ however, no paramagnetic species are involved as endogenous cofactor in such study, as most of the DNAzymes involve, and the authors needed to replace the native cofactor with a paramagnetic species (i.e., Mn^2+^ versus Mg^2+^) in order to use EPR/ESR techniques. A recent computational study on RNA‐cleaving DNA shows also the diverse role played by the metal ion.^[^ ^24^ ^]^

Our integrative approach combining Molecular Dynamics simulations (MD) provide atomistic structural details of the pre‐catalytic conformational properties of the 46mer and advanced the Electron Paramagnetic Resonance (EPR) spectroscopy (also known as Electron Spin Resonance, ESR) reveals information on the intrinsic structure of the DNAzyme and the DNAzyme in action (i.e., during the oxidative cleavage). Mass spectrometry, NMR‐DOSY and MD techniques provide mechanistic and structural features of the global architecture of the DNAzyme, validating one of the putative process implying H‐abstraction on the C‐4′ site.

## 2 Results and Discussion

**Figure 1:**
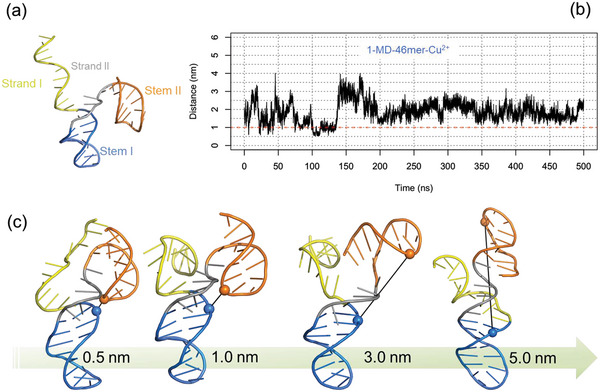
a) Modelled structure of the 46mer for MD simulations without Cu^2+^; strand regions and stem regions are highlighted. b) Time series distance profile between the phosphate atoms of A14 and A41 from the MD trajectory of 46mer with Cu^2+^ (1‐MD‐46mer Cu2^+^). c) Structures from MD ensemble with Cu^2+^, selected based on distance between phosphate atoms of A14 (blue) and A41 (orange) ranging from 0.5 to 5 nm.

A 3D structure of the 46mer DNA was modelled and subjected to MD simulations without (MD‐46mer, **Figure** **1**a) and with Cu^2+^ (MD‐46mer‐Cu^2+^) (Figure S2, Supporting Information). The modeled secondary structure of the 46mer DNA consists of two stem‐loops (I and II) that are interspersed with three single‐stranded domains. This secondary structure is very dynamic because of very high flexibility of the strand I and the stem loops. Such structure agrees with previous essays probing the influence of the length of the catalyst. Indeed, although stem I formation is important for DNAzyme function, the sequences that comprise this structural element can be varied as long as base complementation is retained (i.e., short DNA sequences have been tested in the past to reduce the size of the catalyst 69‐mer and still keep the catalytic activity).^[^ ^16^ ^]^

In Figure 1c the range of selected conformations accessible with Cu^2+^, from the MD ensemble of MD‐46mer‐Cu^2+^ are shown and an extended repertoire of 46mer structures without and with Cu^2+^ are reported in Figures S3 and S4 (Supporting Information). From these ensembles, to study the dynamics of the stem loops. distances between phosphate atoms of selected residue pairs of Stem I and Stem II (A14‐A41 and A18‐A41) were extracted, suggesting a highly dynamic structure of the 46‐mer (Figure S5, Supporting Information). The presence of copper ion(s) shifts the distribution to the left for A14‐A41 (Figure S5a, Supporting Information), as for the RNA‐cleaving 8–17 DNAzyme;^[^ ^24^ ^]^ the conformational polymorphism of the 46mer is further evident from the distances measured between several pairs (C6‐A21 and G19‐C46) chosen to study the dynamic features of the 46‐mer (Figure S5, Supporting Information).

**Figure 2:**
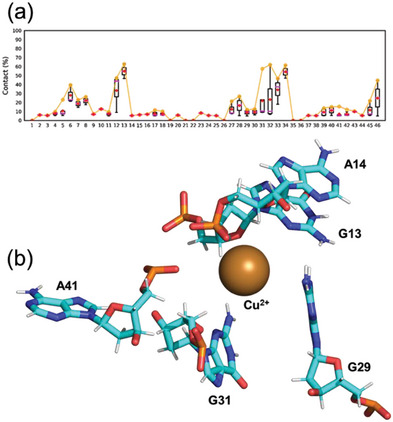
a) Interaction between Cu2^+^ and the 46mer with a distance cut‐off of 0.4 nm was defined as residence time and the contact frequency as percentage of simulation time is shown for 1‐MD‐46mer‐ Cu^2+^ (resident time analysis, RTA). b) Structure from 1‐MD‐46mer‐ Cu2^+^ with a distance of 0.5 nm between A14 and A41, showing a plausible pre‐reactive complex where Cu2^+^ is interacting with residues from substrate and catalytic strands simultaneously.

To study the interaction of Cu^2+^ with the 46mer, contacts between Cu^2+^ and the DNA nucleotides was analyzed with a distance cut‐off of 0.4 nm and residence time analysis (RTA) was defined as the percentage of simulation time Cu^2+^ was within 0.4 nm of the DNA (**Figure** **2**a; Figure S6, Supporting Information). Residues 5–10 and 27–34 and 40–46 had more interaction with Cu^2+^ (≥10% of simulation time) across the entire ten replicas of MD‐46mer‐Cu^2+^.

In replica 1, the shortest distance between A14‐A41 nucleotide pairs (0.5 nm) for ≈20 ns (Figure 1b; Figure S7, Supporting Information) was observed; in this replica interaction between Cu^2+^ and nucleotides C6‐A8, C12, G13, C27, C28, and G31‐T34 for >10% of the simulation time (Figure S7, Supporting Information) can be seen. The conformation with the shortest distance of 0.5 nm between A14 and A41 has a single Cu^2+^ ion that can simultaneously interact with the substrate and the catalytic sites from s4 and c4 strands, with a plausible pre‐reactive complex geometry shown in Figure 2b with interactions between Cu^2+^ and G13, A14, G29, A31, and A41. Excluding replicas 3 and 9 with Cu^2+^, in the rest of the replicas, the shortest distance between A14 and A41 is ≈1 nm suggesting that the electrostatic repulsion between the phosphate backbones of the stem loops can hinder the formation of ideal geometry for the self‐cleavage unless a Cu^2+^ ion is strategically present for the purpose, in agreement with the very slow catalytic rate of ≈0.3 min^−1^ for the 46mer.^[^ ^16^ ^]^

Distance distributions estimated by MD analysis have been complemented by distance distributions obtained by PELDOR/DEER EPR techniques,^[^ ^25^, ^26^ ^]^ used on the c4s4 architecture, labeled with nitroxide spin probes (Figure S8, Supporting Information).^[^ ^27^, ^28^ ^]^ The different populated states of c4s4 conformers detected by dipolar spectroscopy support the scenario obtained by MD; the high flexibility typical of such artificial enzymes is confirmed also for the c4s4 architecture (Figure S9, Supporting Information).

**Figure 3:**
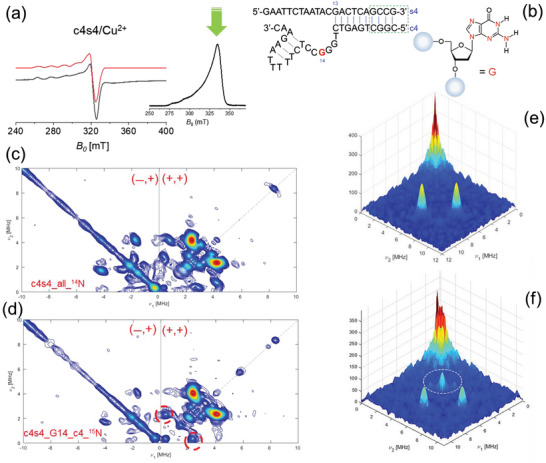
a) CW‐EPR spectra for Cu‐c4s4 sample at X‐Band, measured at 120 K. The red dotted lines are the fitting of the experimental spectra (black line). On the right the Echo‐Detected Field‐Swept (EDFS) spectrum; the green arrow indicates the selected observed field for the 2D‐ESEEM experiment. b) Structure of the c4s4 complex with the isotopic labeled by ^15^N at Guanosine residue‐14 of the catalyst (c4). c,d) HYSCORE (HYperfine Sublevel COrRElation spectroscopy) 2D EPR experiments recorded at 9.72 GHz for the native c4s4 architecture containing Cu^2+^ and for the isotopically labeled structure depicted in b. By comparing the (+/+) quadrant of the HYSCORE experiments for the unlabeled c4s4 with the samples containing guanosine G14 (c4) sequence additional cross peaks with Δ*v* = 2.7 *MHz* have been observed. These additional cross peaks are generated by the hyperfine coupling Cu^2+/15^N (having ^15^N nuclear spin I = 1/2, the manifold of the coupling generating a doublet without the presence of double quanta transition and/or frequencies combination). ^15^N Larmor frequency (1.51 MHz) is indeed observed only on the HYSCORE experiments recorded on the sequences containing isotopologues (circled cross peaks. In addition, the comparison between the (‐/+) quadrant of the unlabeled/labeled samples shows the reduced cross peaks (double‐quanta transition for ^14^N). e,f) 3D plot for the HYSCORE spectra recorded at Q‐band (34 GHz) for (*top*) c4s4 unlabeled sample and for the c4s4 isotopically labeled (^15^N) (*bottom*) at Guanosine residue‐14 of the catalyst (c4).

Short‐range interactions between c4s4 and Cu^2+^ ions have been studied in order to provide the coordination sphere of the paramagnetic center. As for copper chlorine in frozen solution and for monomeric ligand/Cu^2+^ complex previously described,^[^ ^29^ ^]^ spectral features typical for Cu^2+^ complexes with a (d_x_^2^__y_^2^) ground state (g_ǁ_ > g_┴_) are observed (**Figure** **3**a).^[^ ^30^ ^]^ The comparison of the g values and the Cu^2+^ hyperfine parameters between Cu^2+^ chlorine and c4s4 adduct indicates a metal–nucleobase interaction. The EPR parameters of Cu^2+^ chlorine are typical from Cu^2+^ complexes with four oxygen atoms as equatorial ligands.^[^ ^30^ ^]^ Instead the g values and Cu^2+^ hyperfine parameters of Cu^2+^/c4s4 complex are typical for Cu^2+^complexes with either three oxygen atoms or one nitrogen atom. Furthermore, the g values and the hyperfine parameters of samples containing c4s4 (all ^14^N) and c4s4 (selectively labeled with ^15^N) are very similar to each other (Figure S10 and Table S1, Supporting Information). This suggests the presence of interactions between the Cu^2+^ ion and nucleobase(s).^[^ ^31^ ^]^

Although the CW EPR data provides evidence of a coordination of the Cu^2+^ metal by c4s4 systems, detailed information about the metal ion site cannot be obtained by 1D‐CW experiments. Thus, further insights into the metal ion site are provided by 2D‐pulsed EPR techniques. To get information about the weakly coupled nuclei, the 2D‐ESEEM (known as HYSCORE) technique has been used. In Figure 3a inset, the echo‐field swept spectrum and the observed field (green arrow) chosen for the HYSCORE experiment is shown. Hyperfine spectroscopy has been supported by the use of isotopologues on selected nucleotides (Figure 3a, *right*). The choice of isotopically enriched residues has been driven by the RTA analysis from MD, in order to exploit the potentiality of HYSCORE, despite the excess of Cu^2+^ cofactor. The HYSCORE 2D‐spectrum of c4s4 bimolecular complex formed with Cu^2+^ (Figure 3c) confirms *a plethora* of weak coupling with ^14^N nuclei within the base‐pair arrangement. Based on the RTA from MD simulations, ^15^N isotopic labeled nucleobases, G14 on the catalyst, have been used to “switch on” the selective coupling suggested in the previous MD section.

The HYSCORE spectrum of the Cu^2+^/c4s4 complex also shows two types of weakly coupled protons. One type is characterized by an intense ridge close to the antidiagonal at the proton Larmor frequency (14 MHz) and can be assigned either to protons of water molecules coordinated in axial positions or/and to weakly coupled protons of solvent molecules.^[^ ^30^ ^]^ (Figure S11, Supporting Information). A more crowded scenario is reserved to the ^14^N hyperfine coupling spectral regions; as the MD analysis has suggested, several sites are coordinated to Cu^2+^, involving several ^14^N nuclei. The low‐frequency regions of the HYSCORE spectra of samples c4s4 and c4s4 (G14‐c4‐^15^N) are reported in Figure 3c,d. The HYSCORE spectrum of Cu^2+^/c4s4 (all‐^14^N) is dominated by cross‐peaks that are assigned to double‐quantum (DQ) correlation peaks from ^14^N. Furthermore base‐pairing around the copper coordination increases the number of ^14^N nuclei, as can be observed in the HYSCORE experiments. As previously reported for guanosine mono‐phosphate,^[^ ^30^ ^]^ also quadrupole interaction can be considered (|e^2^qQ/h| = 3.02 ± 0.05 MHz) (Figure S12, HYSCORE fitting at X‐band and Table S2, Supporting Information). Even if those samples contain an excess of cofactor (Cu^2+^) cross peaks for ^15^N (as doublet) are successfully observed. Indeed, the assignment to the remote nitrogen is reinforced by the analysis of c4s4 containing isotopologues (^15^N) and a similar remote nitrogen pattern is obtained in addition to cross‐peaks derived by interaction with ^14^N nuclei. Furthermore, within the base‐pair arrangement, it is possible to discard N1, N2 and N3 of deoxyguanosine within the coordination sphere; those are involved within the hydrogen bonding network of the minor groove of DNA duplex form and oriented far from the metal center. Furthermore, N7 is more strongly coordinated (not detectable by HYSCORE); thus, the observed hyperfine coupling can be assigned to the N7 atom. The HYSCORE spectrum at 9.7 GHz (X‐band) is characterized by single‐quantum, double quantum, and combination peaks of nitrogen nuclei. Cross‐peaks related to ^15^N are observed for the G14 on the catalyst (c4 for the complex c4s4, numbered at G31 on the 46‐mer) and on G13 (substrate). To discard any ambiguities, HYSCORE experiments have been recorded at higher frequency/field (34 GHz, Q‐band). Those experiments confirmed the coupling of Cu^2+^ with ^15^N on the guanine of the catalyst with higher resolution because of cancellation conditions for part of nitrogen nuclei (Figure 3e,f). Preliminary DFT calculations on a simplified model (G13/G41 of the 46mer) could estimate hyperfine couplings with a good agreement with the EPR data (Figure S13 and Tables S3–S5, Supporting Information).

In addition to local interactions deciphered by combining MD analysis and EPR/ESR 1D and 2D techniques, mass spectrometry and NMR diffusion‐ordered spectroscopy (DOSY) allowed us to validate the products of the cleavage reaction as well as radical intermediates. In particular, hydroxyl radical generated by a Fenton‐like reaction^[^ ^32^ ^]^ has been detected by spin‐trap technique (Figure S15, Supporting Information); the products of cleavage reaction (3′‐phosphoglycolate and 5′‐phosphate fragments) confirmed by NMR diffusion (DOSY) (Figures S16–S18, Table S6, Supporting Information ) and MALDI‐TOF MS experiments able to discard putative hydrogen abstraction (Figures S19–S29, Supporting Information).

## 3 Conclusions

In this first study, we have thus identified some key‐points DNAzyme activity: the snapshot from the MD simulation of MD‐46mer‐Cu^2+^ from replica 1, at 104.65 ns with distance of 0.53 nm between A14 and A41, (Figure 2b) suggests the assignment to a “pre‐reaction complex”, with a single Cu^2+^ interacting with both the substrate, G13, and the catalytic arm G31 simultaneously. Such structure gives insights into the specificity of the binding, as confirmed by the EPR data. If EPR hyperfine spectroscopy has been established as method of choice for “single‐site” metal ‐based enzymes (i.e., Fe‐S cluster proteins),^[^ ^33^ ^]^ mechanistic insights into DNAzymes are strongly affected by multiple binding sites.^[^ ^25^ ^]^ The combined approach (EPR/MD/NMR/MS) can focus on the paramagnetic species in order to identify the role of the copper ion(s) both as structural (for the coordination on the catalytic core) and as mechanistic (for the oxidative step)^[^ ^34^ ^]^ as far as hydrogen abstraction on the C4’ is involved.

Those roles identified by 2D hyperfine spectroscopy agree with a fitting of EPR/ESR binding isotherms recently proposed for the manganese ions^[^ ^8^, ^35^ ^]^ and with the few cooperatively binding modes proposed for the DNAzymes.^[^ ^36^, ^37^, ^38^, ^39^ ^]^ The cooperative binding is combined with the intrinsic flexibility of the c4s4 (46mer) architecture.^[^ ^40^, ^41^ ^]^

The EPR/ESR spectroscopic protocol here proposed combined with MD analysis could point out both mechanistic and structural features unknown to date. By deciphering mechanistic features, this work can contribute to design a next generation of ‘DNA‐cleaving DNAzymes’. Those novel structures should exhibit a) substrate specificity with minimal demands on the sequence of the target DNA, b) a rapid and multiple turn‐over kinetics under mild reaction conditions. Furthermore, exploring the reaction mechanism using QM/MM simulations, with assessment using atomistic structural studies would a very exciting future avenue. Additionally, this work paves the way for increasing complexity both for paramagnetic cofactors (i.e., metal and lanthanides) and for structural features (i.e., DNAzymes containing several branched regions). Ligation reactions of RNA and/or DNA strands involving radical paths are suitable candidates for widening the approach presented in this work.

## Conflict of Interest

The authors declare no conflict of interest.

## Supporting information

Supporting Information

## Data Availability

The data that support the findings of this study are available from figshare (https://doi.org/10.17633/rd.brunel.23522544.v1) and in the supplementary material of this article.
