# Supplementary material for: Cleaving DNA with DNA: Cooperative Tuning of Structure and Reactivity Driven by Copper Ions
Source: Adv Sci (Weinh). 2024 Feb 28;11(16):2306710. doi: 10.1002/advs.202306710 (PMC11040348; doi:10.1002/advs.202306710)
Supplement: Supplementary file 1 — Supporting Information [file ADVS-11-2306710-s001.pdf]

ADVANCED SCIENCE
Open Access

Supporting Information

for *Adv. Sci.*, DOI 10.1002/advs.202306710

Cleaving DNA with DNA: Cooperative Tuning of Structure and Reactivity Driven by Copper Ions

Sarath Chandra Dantu, Mahdi Khalil, Marc Bria, Christine Saint-Pierre, Maylis Orio, Didier Gasparutto and Giuseppe Sicoli**

SUPPLEMENTARY INFORMATION

Cleaving DNA with DNA: cooperative tuning of structure and reactivity driven by copper ions

Sarath Chandra Dantu,[a] Mahdi Khalil,[b] Marc Bria,[c] Christine Saint-Pierre,[d] Maylis Orio,[e] Didier Gasparutto,[d] Giuseppe Sicoli[b]*

[a] Dr. S. C. Dantu
Computational Biology, Department of Computer Science
Brunel University London
Kingston Lane, Uxbridge UB8 3PH, United Kingdom

[b] M. Khalil, Dr. G. Sicoli
LASIRE
CNRS UMR 8516, University of Lille
C4 building, Avenue Paul Langevin, F-59655, Villeneuve d'Ascq, France
Email : giuseppe.sicoli@univ-lille.fr

[c] M. Bria
Michle-Eugène Chevreul Institute
FR 2638
Avenue Paul Langevin, F-59655, Villeneuve d'Ascq, France

[d] C. Saint-Pierre, Dr. D. Gasparutto
UMR 5819 INAC-SyMMES-CREAB
Grenoble University, CEA-CNRS-UGA
Avenue des Martyrs, F-38000 Grenoble, France

[e] Dr. Maylis Orio
Aix Marseille Université, CNRS
Centrale Marseille, iSm2, UMR CNRS 7313
13397 Marseille, France

*Corresponding authors: sarath.dantu@brunel.ac.uk, giuseppe.sicoli@univ-lille.fr

Table of contents

S1. Materials and Methods

Figure S2. Modelled structure of 46-mer with Cu2+ randomly placed around the simulation box

Figure S3. Cluster representatives and the respective population percentage of the cluster from the MD ensemble of 46-mer without Cu^{2+}

Figure S4. Cluster representatives and the respective population percentage of the cluster from the MD ensemble of 46-mer with Cu^{2+}

Figure S5. Distance distributions profiles obtained from MD ensembles with (MD-46mer-Cu^{2+}) and without Cu^{2+} (MD-46mer) between the phosphate atoms of C6 and A21 and G19 and C46.

Figure S6. Interaction between $Cu2^{+}$ and the 46mer with a distance cut-off of 0.4nm was defined as residence time (RTA) and the contact frequency as percentage of simulation time is shown for each independent MD trajectories below

Figure S7. Time series distance profiles between the phosphate atoms of A14 and A41 calculated from the independent trajectories of MD simulations with $Cu2^{+}$ (MD46mer- $Cu2^{+}$)

Figure S8. Spin labeled c4s4 oligomer and structures of nitroxide spin labels

Figure S9. Distance distributions from DEER/ PELDOR

Figure S10. CW EPR spectra and corresponding EasySpin fit; Peisach Plot and Table S1 summarizing EPR parameters

Figure S11. Full HYSCORE spectrum for the c4s4

Figure S12. HYSCORE and Hyscorean fit, Table S2

Figure S13. DFT-model for CopperG13G31 obtained from geometry optimization

S14. Table S3 and Table S4 summarizing g-factor and hyperfine data

Figure S15. Spin trap (DMPO)

S16. Diffusion coefficients summarized in Table S5

Figure S17. Plot of log(D) versus log(MW); diffusion coefficients/1H-NMR spectrum

Figure S18. a) NMR spectrum (900 MHz) monomeric guanosine; b) NMR spectrum (900 MHz) monomeric guanosine with Cu^{2+}; c) NMR spectrum (900 MHz) c4s4; d) NMR spectrum (900 MHz) c4s4 with Cu^{2+}

Figure S19. MALDI-TOF of the cleaved c4s4

Figure S20. Putative mechanism on C4' hydrogen abstraction

Figure S21. Putative mechanism on C1' hydrogen abstraction

Figure S22. Putative mechanism on C5' hydrogen abstraction

Figure S23. MALDI-TOF on the c4s4 double-strand

Figure S24. MALDI-TOF on the cleaved c4s4

Figure S25. MALDI-TOF control experiment on s4

Figure S26. MALDI-TOF control experiment on c4

Figure S27. Products of cleavages and corresponding molecular weight

Figure S28. MALDI-TOF (zoom) on the peak 5240.8 m/z

Figure S29. Scheme of enzymatic digestion on the fragment 5240.8 m/z

Figure S30. Identification of the fragment 5240.8 m/z as product of catalyst decomposition

Supporting Information S1. MATERIALS AND METHODS

Sample preparation for EPR experiments. Duplexes have been formed by heating DNA strands at 90°C for 3 minutes and slowly cooled down to room temperature. For the different pH, three buffers were used: Sodium Acetate (pH 4.00), Cacodylate (pH 7.45), Glycine/NaOH (pH 10.3). 10% (v/v) of glycerol was added before freezing the sample in liquid Nitrogen. With respect to Cu^{2+} solution ($CuCl_2$), a molar excess of monomeric ligand or double helix was used, up to an excess of 1:10. 100 mL solutions were used in 4 mm tubes (X-band) and 3 mm tubes (Q-band), respectively.

EPR experiments. Continuous Wave (CW) X-Band measurements were carried out using an X-band Bruker E500 instrument (9.4 GHz, TE_{012} resonator) equipped with a nitrogen flow cryostat. All CW experiments were recorded at 120 K and with a shot repetition rate of 100 Hz, unless stated otherwise. Pulsed EPR experiments at X-band were performed on a Bruker ELEXYS E-580 X-band spectrometer with a SuperX-FT microwave bridge and a Bruker ER EN4118X-MD4 dielectric resonator. Cryogenic temperatures (20 K) were obtained by the use of an Oxford flow cryostat. The field-swept EPR spectra were recorded by electron spin echo (ESE) detection; electron-spin-echo (ESE)-detected EPR experiments were carried out with the pulse sequence: π/2–τ–π–τ–echo. For the X-band experiments the mw pulse lengths $t_{π/2}$ = 16 ns and $t_π$ = 32 ns and a τ value of 200 ns were used. A two-step phase-cycle was applied to remove all unwanted echoes. The Hyperfine Sublevel Correlation (HYSCORE) experiments were carried out using the pulse sequence π/2–τ–π/2–t_1–π–t_2–π/2–τ–echo. The time traces of the HYSCORE spectra were baseline corrected using a third-order polynomial, apodized with a Hamming window and zero-filled. After two-dimensional Fourier transformation, the absolute value spectra were calculated. A four-step phase cycle (for X-band experiments) was used to remove unwanted echoes. The pulse sequence for the four-pulse DEER experiment was $π/2_{obs} − τ_1 − π_{obs} − t_1 − π_{pump} − (τ_1 + τ_2 − t_1) − π_{obs} − τ_2$. The pump pulse was applied on the spectral maximum and the observer pulses were applied at a frequency offset of 55 MHz. Measurements on the Bruker Elexsys E580 spectrometer were acquired with a pulse delay $τ_1$ of 200 ns and a dead time delay of 100 ns. All DEER data were analyzed using DeerAnalysis2022 based on MATLAB. The distance distribution $P_{dis}(r)$ was fitted by Tikhonov regularization (using residual method for regularization parameter selection). The capture of active radicals generated during the reaction was examined by EPR spectra (Bruker ELEXSYS 500 spectrometer) using DMPO as a spin trapping agent (Supplementary methods). DMPO (0.8 M) was immediately added to the c4s4 solution and transferred to a glass capillary tube.

Then, the capillary tube was placed into a quartz EPR tube and EPR spectra were recorded. Typical spectrometer parameters are shown as follows, scan range: 100 G; center field set: 3510 G; time constant: 1.25 ms; scan time: 40.96 s; modulation amplitude: 0.5 G; modulation frequency: 100 kHz; receiver gain: 1.00 × 103; microwave power: 19.17 mW. Signal fitting was carried out using the Spin Fit program (Bruker). Signal fitting for HYSCORE experiments has been carried out using the Hyscorean software (https://epr.ethz.ch/software.html).

Modelling and MD simulations. The 3D structure of the 46-mer (Fig. 1a) was modelled using x3dna package (v2)[42] using 1JVE pdb[43] as template for the "GGA hairpin". Parmbsc1 forcefield[44] was used for the MD simulation of the 46mer using gromacs2016 package[45] 46-mer was placed in a dodecahedron box with 1.0 nm distance between the box walls and the DNA molecular and was solvated with tip3p[46] water molecules (MD-46mer). For simulations with Cu2+ ions, 10 mM of Cu2+ ions were randomly placed in the simulation box (MD-46mer-Cu^{2+}) (Fig. S2). Simulation system was energy minimized using steepest descent algorithm until the largest force was smaller than 1000 kJ/mol/nm, followed by temperature equilibration to 300 K in 100ps using Berendsen thermostat with a tau-t of 0.2 ps. Pressure was equilibrated to 1 atm in 1 ns using Berendsen barostat and temperature was regulated using Berendsen thermostat at 300 K.[47] Production run simulations were started using the equilibrated structures and ten replicas were simulated for 500 ns. For production run simulations, temperature was regulated usi velocity-rescaling thermostat[48] and pressure with Parrinello-Rahman barostat[49] at 300 K and 1 atm using tau-t of 0.1ps and tau-p of 2ps. Structures were saved every 10 ps. Clustering of the MD simulations was done using gromos algorithm[50] with snapshots sampled at 200 ps intervals using only phosphate backbone atoms with a RMSD cut-off of 0.2 nm. All the analysis was done using in-house python scripts and data visualization was done using Pymol.[51]

NMR experiments. NMR experiments[52-57] were recorded with a 3-mm NMR sample tube at 298°K using the following Bruker spectrometers with *z*-axis pulsed field gradients: Avance NEO at 900-MHz (21 Tesla) proton frequency with CPTCI CryoProbe is a proton-optimized triple resonance NMR 'inverse' probe. A standard pulse sequence lebpgp2s from Topspin 4.1, (Bruker) was used for diffusion experiments on the 900-MHz (21 Tesla) instrument. In total, 65 536 points with 16 scans were recorded in the proton dimension for each one dimension with variable diffusion gradient strength ranging between 2 and 95% in various steps. The following parameters were used: diffusion time (Δ) 0.085 s, gradient pulse (δ) 2500 µs smoothed rectangular-shaped gradients SMSQ10.100, relaxation delay (d1) 10 s. NMR experiments were recorded with a 5-mm NMR sample tube at 298°K Avance NEO at 400 MHz (9.4Tesla)

equipped with a TBI probe. All spectra were processed with Topspin 4.1(Bruker) and DOSY treatment Software Dynamic center (Bruker)

MALDI-TOF experiments. The MALDI-TOF[58-60] mass spectra measurements were performed in the negative mode on a Microflex mass spectrometer (Bruker, Wissembourg, France). Prior to mass analysis, oligonucleotides solutions were purified and concentrated using Zip Tip pipette tips (Merck Millipore) filled with 0.6 µL C18 resin. A mixture of the purified DNA sample (10 pmol, 1 µL) was added to the matrix (3-hydroxypicolinic acid in 10 mM ammonium citrate buffer) and spotted on a polished stainless target plate using the dried droplet method. Spectra were calibrated using reference oligonucleotides of known masses. Each spectrum were obtained by summing 300 shots by the use of a 337 nm pulsed nitrogen laser beam (60 Hz). Linear mode was run with optimized voltages for ion sources (IonSource-1: 20 kV, IonSource-2: 18.5 kV) and pulsed ion extraction delay was fixed at 100 ns. In order to eliminate the intense low masses of the spectra (matrix peaks, solvents clusters) which normally saturates microchannel plate detectors, and with the aim to enhance the ratio signal to noise, all ions with less mass than 1800 Daltons were deflected. Spectra were accumulated by FlexControl Software (v.3.3.108.0) and processed with FlexAnalysis using Savitsky-Golay algorithm (with 0.2 m/z, one cycle) and baseline subtraction (Top Hat).

DFT calculations. All calculations were performed using the ORCA program package.[61] Full geometry optimization was carried out for using the GGA functional BP86[62] with the def2-TZVP basis sets.[63] For the Coulomb fitting the def2/J auxiliary basis sets were used.[64] For according to the experimental conditions, all calculations were performed using an implicit solvation model (epsilon = 20, refractive index = 1.33)[65] by invoking the Control of the conductor-like polarizable continuum model (CPCM).[66] EPR parameters, namely *g*-tensors and metal hyperfine coupling constant, were obtained from single-point calculations using the previously defined aug-cc-pVTZ-Jmod basis set[67] for Cu, the EPR-II basis for nitrogens[68] while the def2-TZVP basis sets[63] were used for all other atoms. Hyperfine tensors were computed with the B3PW91 functional,[69] which was shown to be the best functional for copper hyperfine coupling constants[67] while the *g*-tensor was computed using a modified version of B3PW91 with 40% exact (Hartree–Fock) exchange.[70]

Supporting Information S2

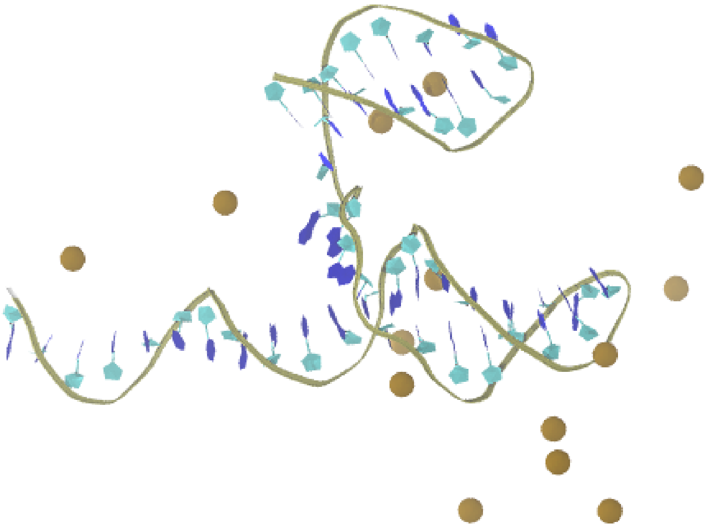

Figure S2. Modelled structure of 46-mer with Cu2+ randomly placed around the simulation box

Supporting Information S3

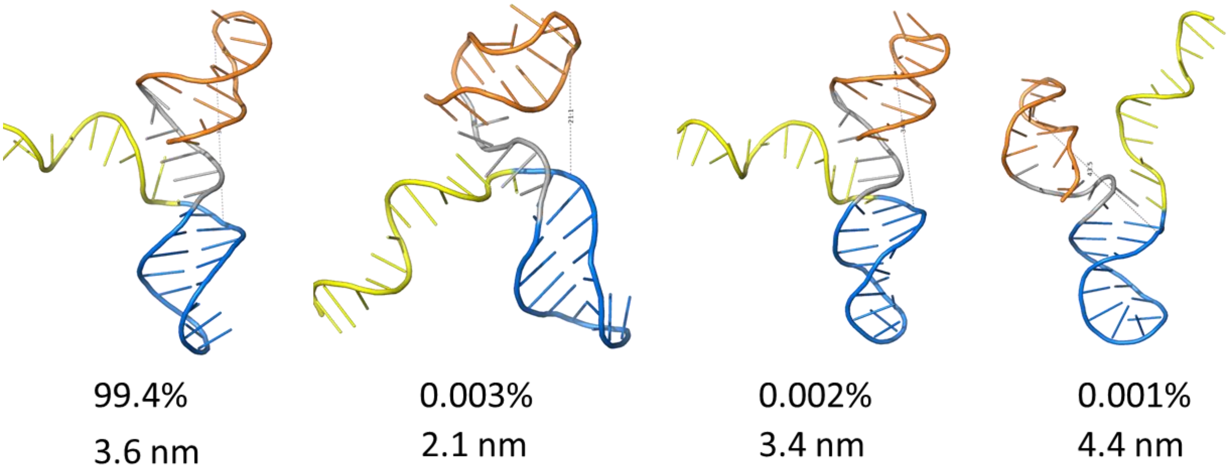

Figure S3. Cluster representatives and the respective population percentage of the cluster from the MD ensemble of 46-mer **without** Cu^{2+}

Supporting Information S4

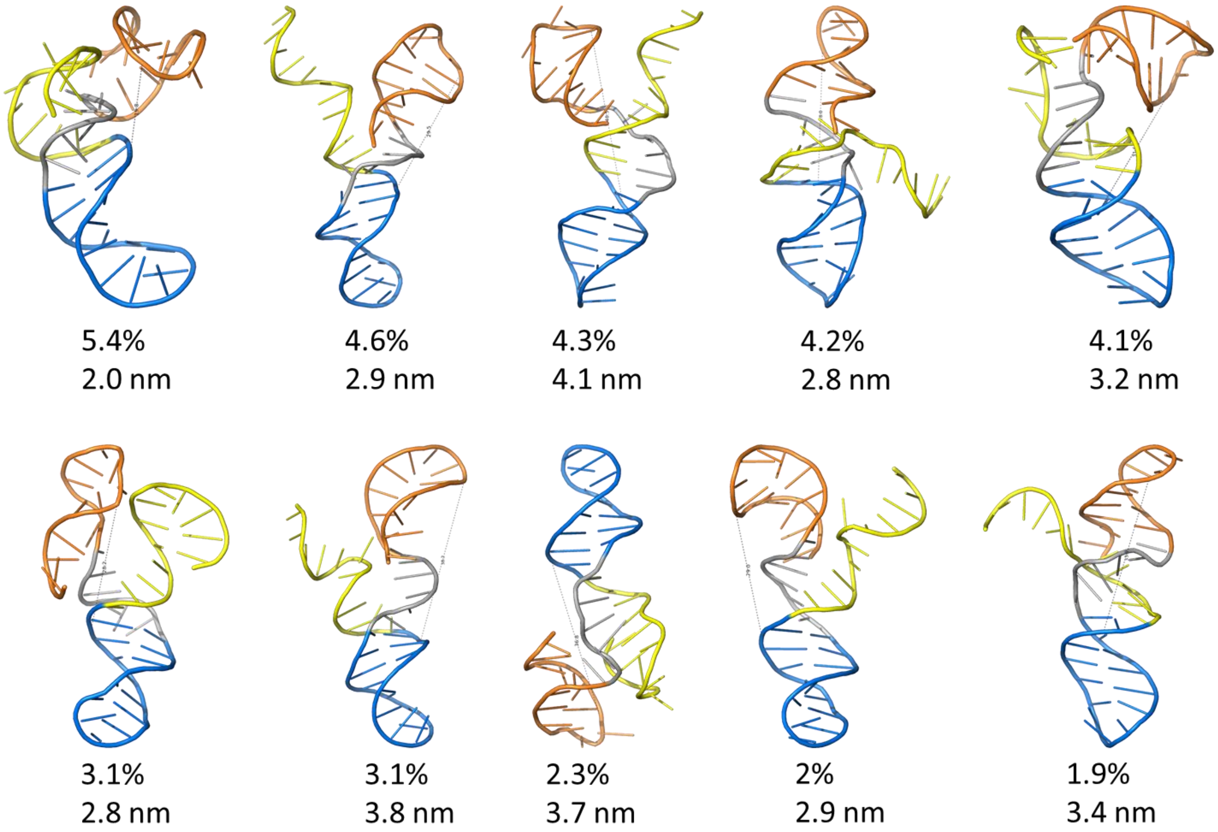

Figure S4. Cluster representatives and the respective population percentage of the cluster from the MD ensemble of 46-mer **with** Cu^{2+}

Supporting Information S5

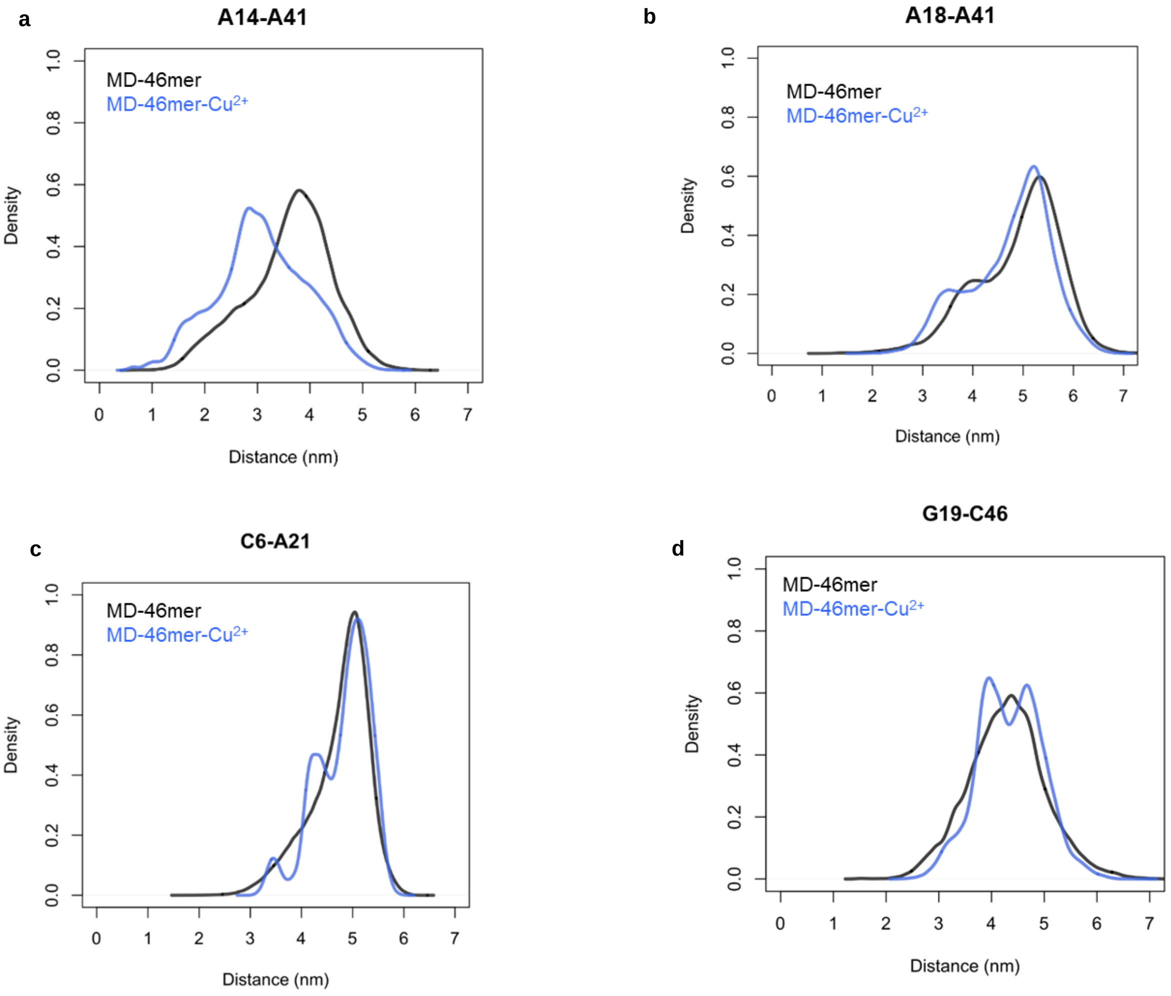

Figure S5. Distance distributions profiles obtained from MD ensembles with (MD-46mer-Cu^{2+}) and without Cu^{2+} (MD-46mer) between the phosphate atoms of a) A14 and A41, b) A18 and A41, c) C6 and A21 and d) G19 and C46.

Supporting Information S6

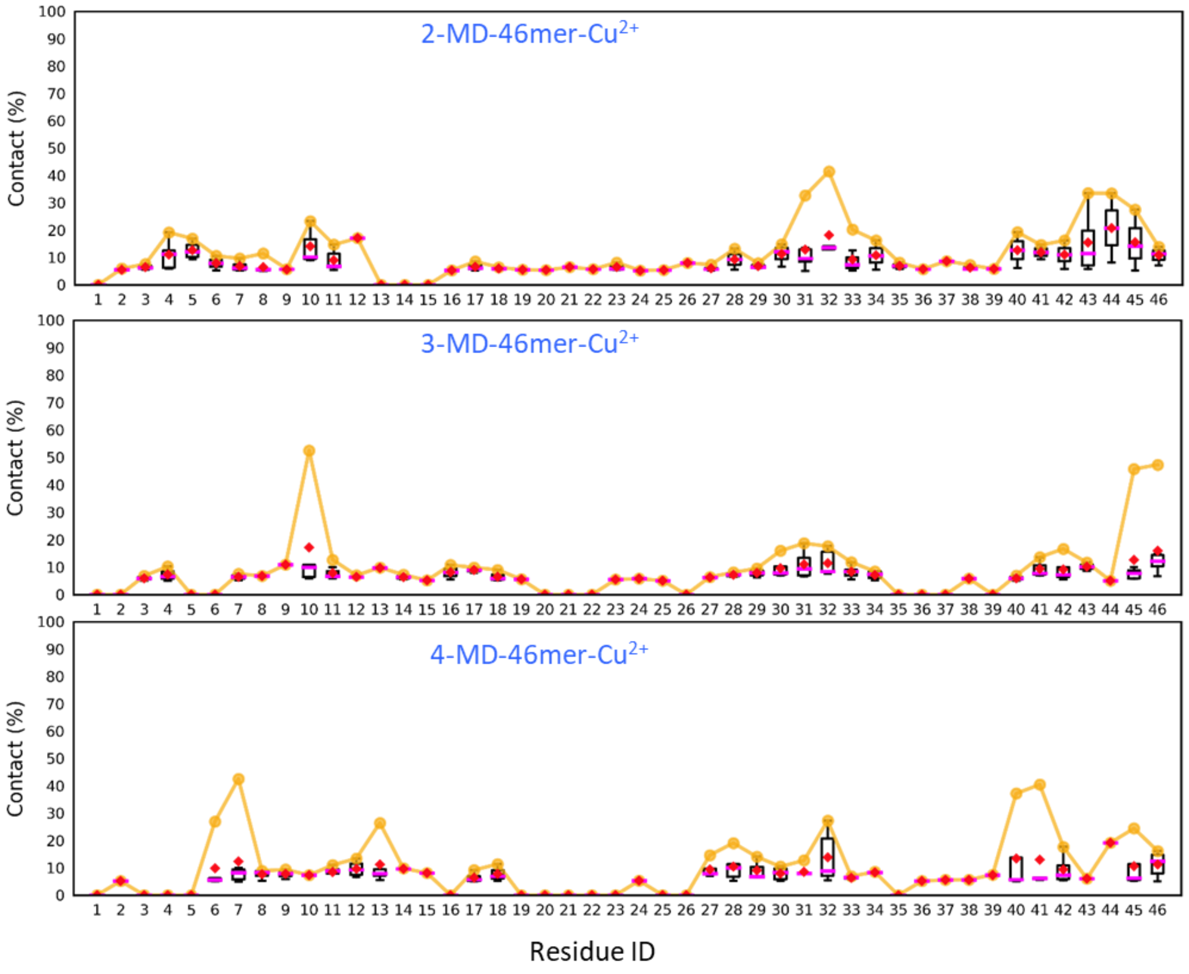

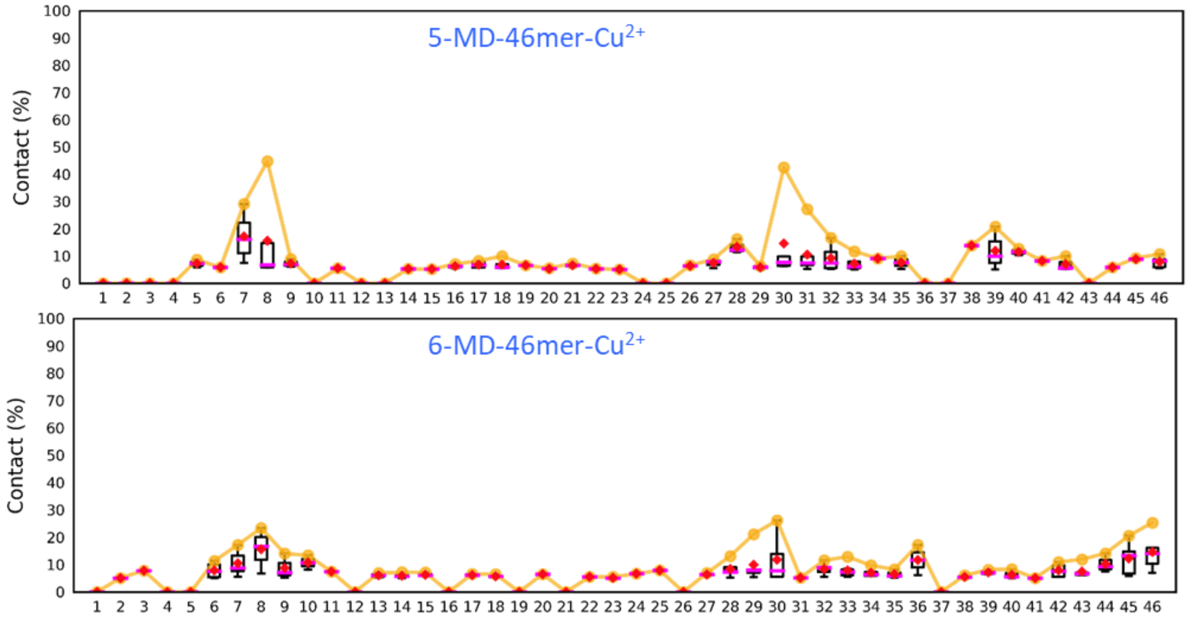

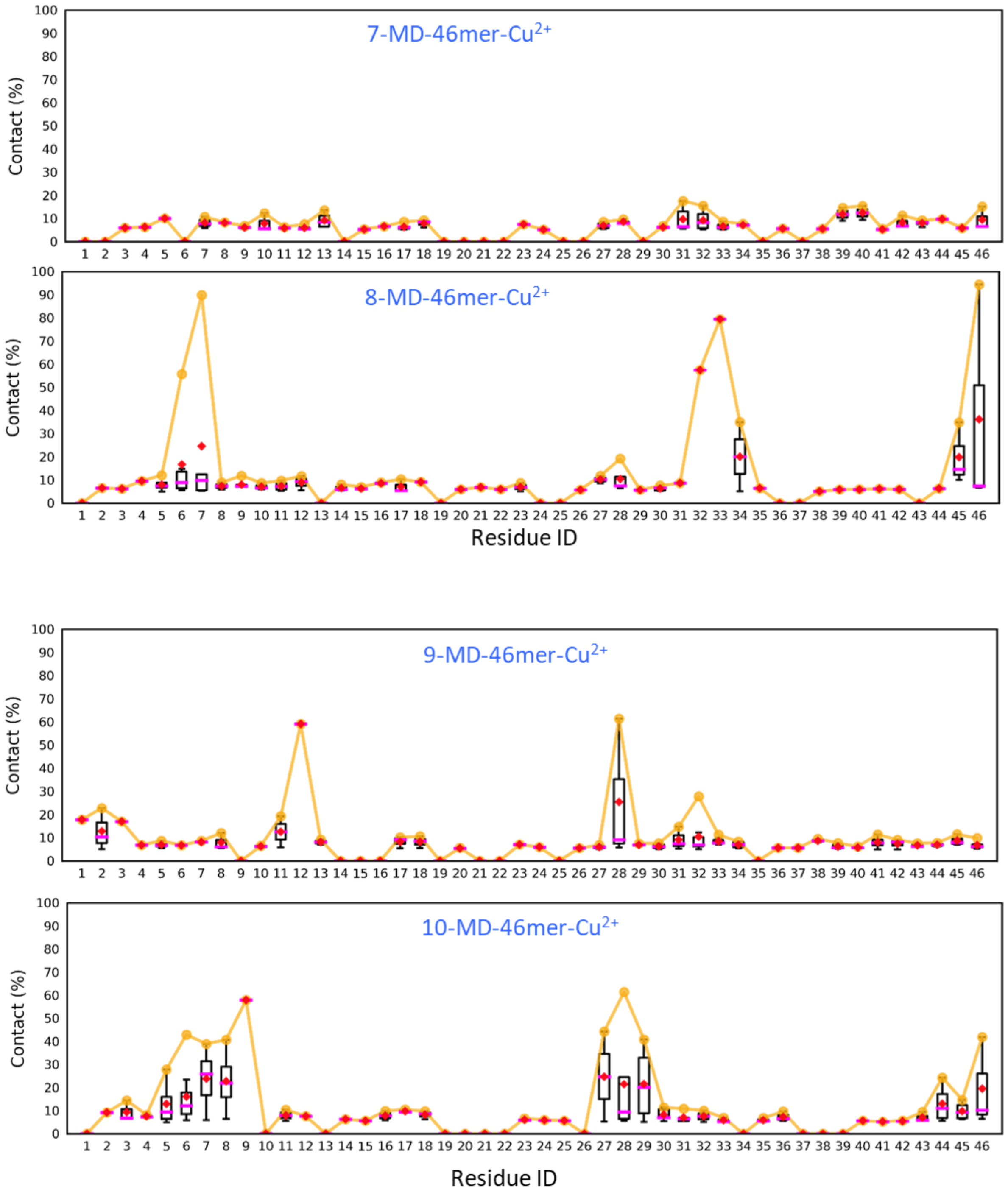

Figure S6. Interaction between Cu2+ and the 46mer with a distance cut-off of 0.4nm was defined as residence time analysis (RTA) and the contact frequency as percentage of simulation time is shown for each independent MD trajectories above.

Supporting Information S7

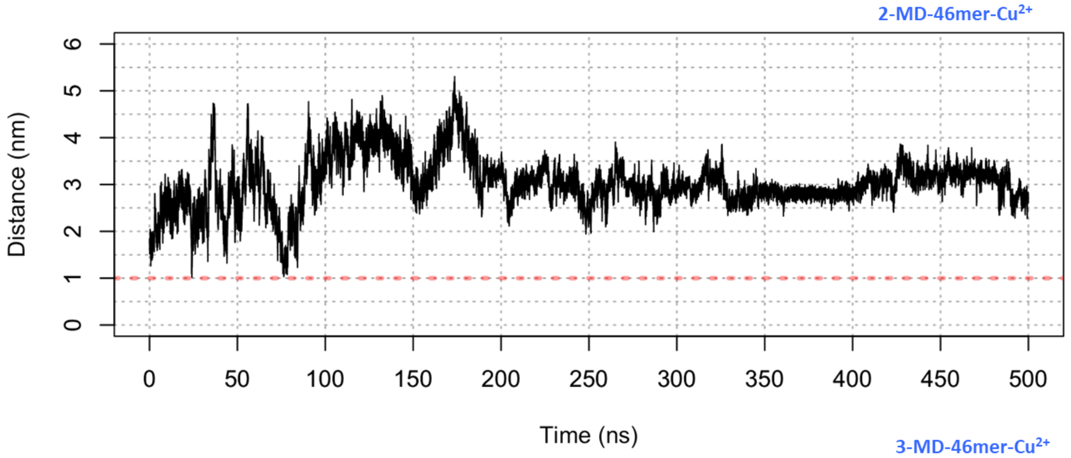

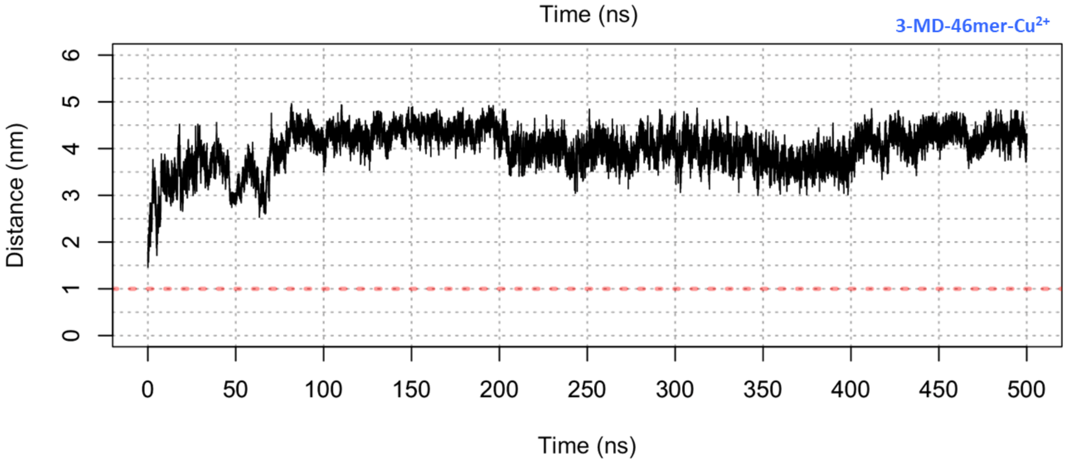

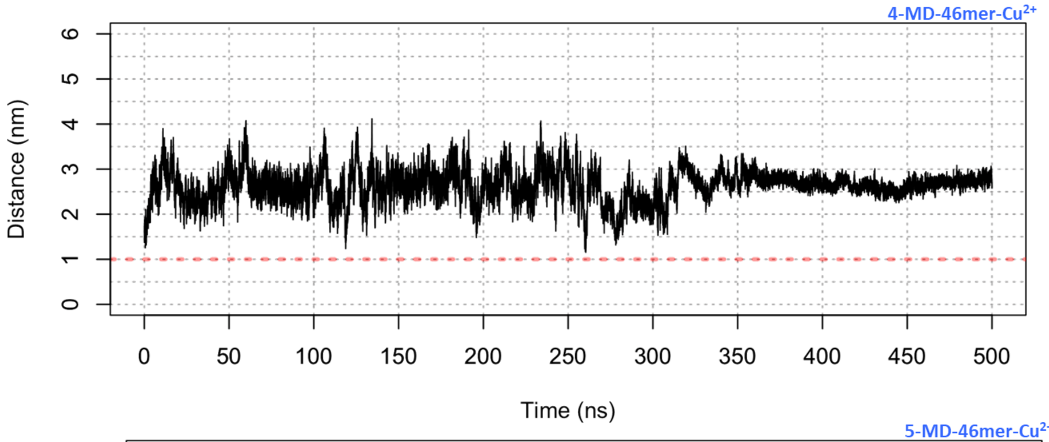

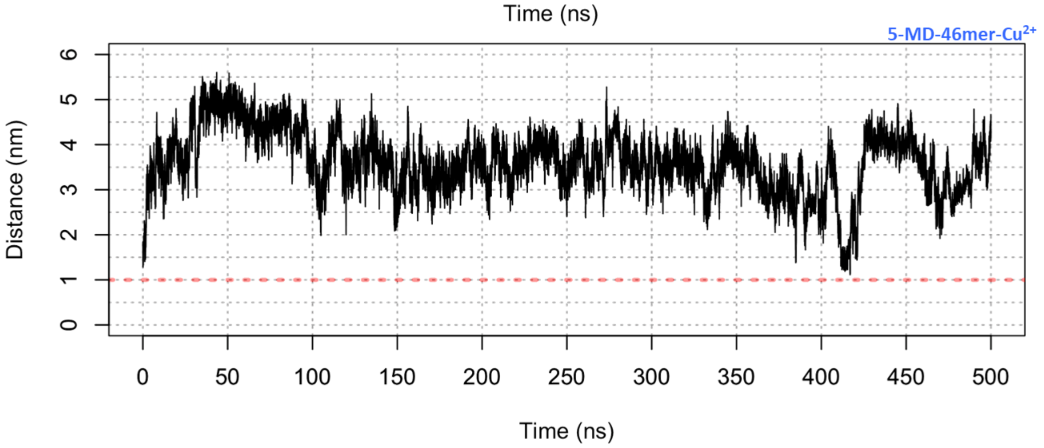

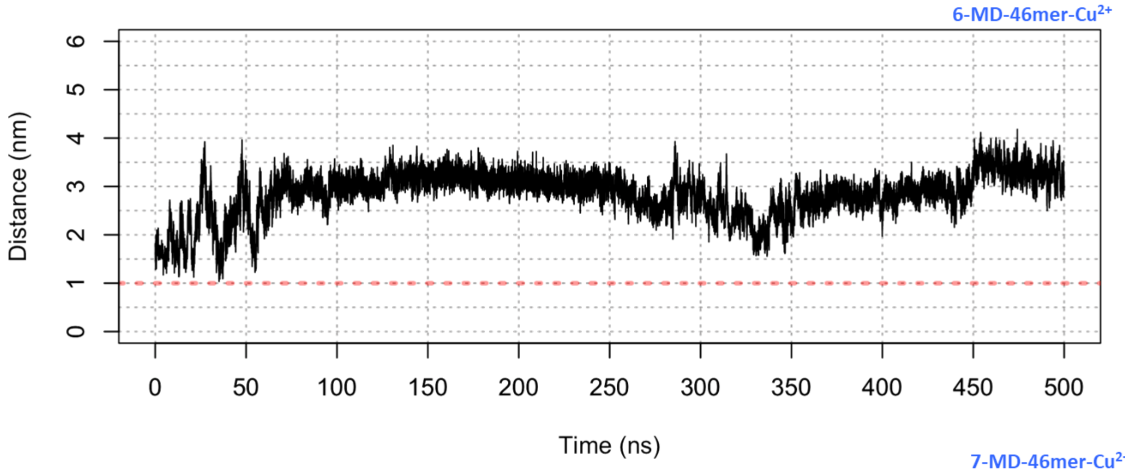
6-MD-46mer-Cu2+
Distance (nm)
Time (ns)

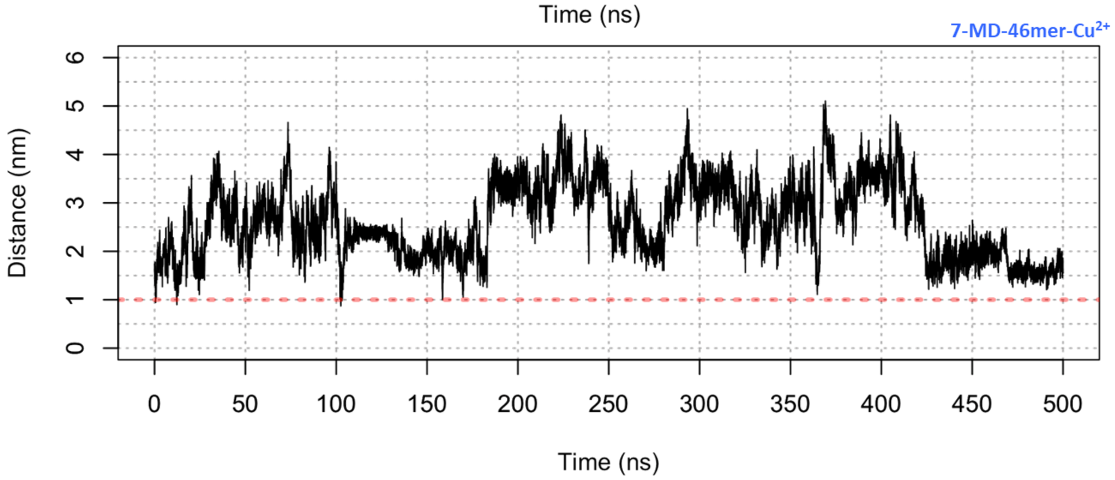
7-MD-46mer-Cu2+
Distance (nm)
Time (ns)

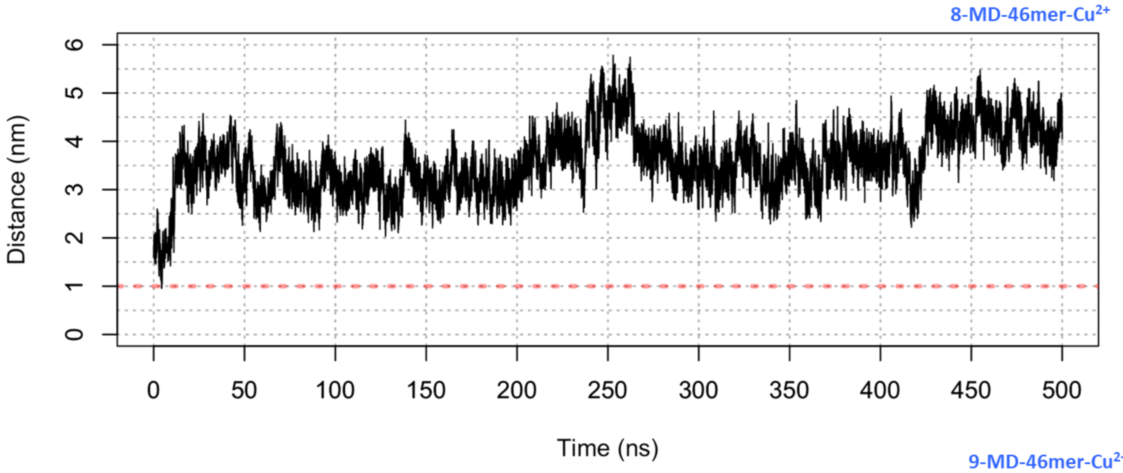
8-MD-46mer-Cu2+
Distance (nm)
Time (ns)

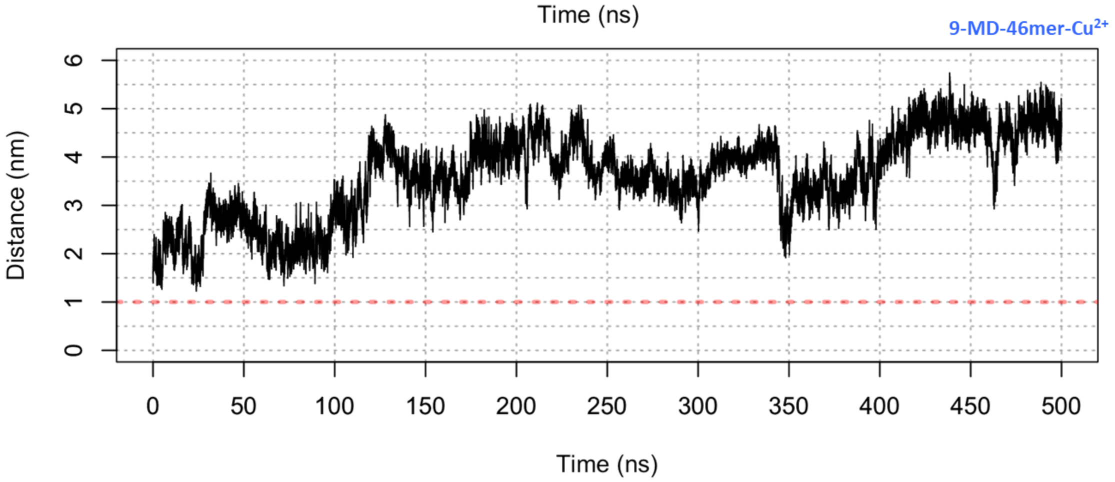
9-MD-46mer-Cu2+
Distance (nm)
Time (ns)

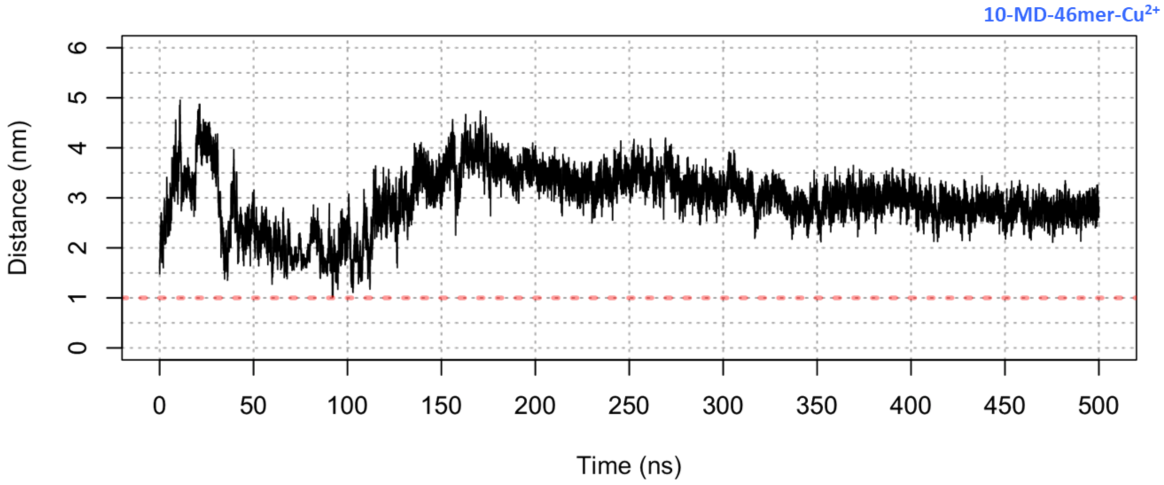

Figure S7. Time series distance profiles between the phosphate atoms of A14 and A41 calculated from the independent trajectories of MD simulations with Cu2^{+} (MD46mer- Cu2^{+}).

Supporting Information S8

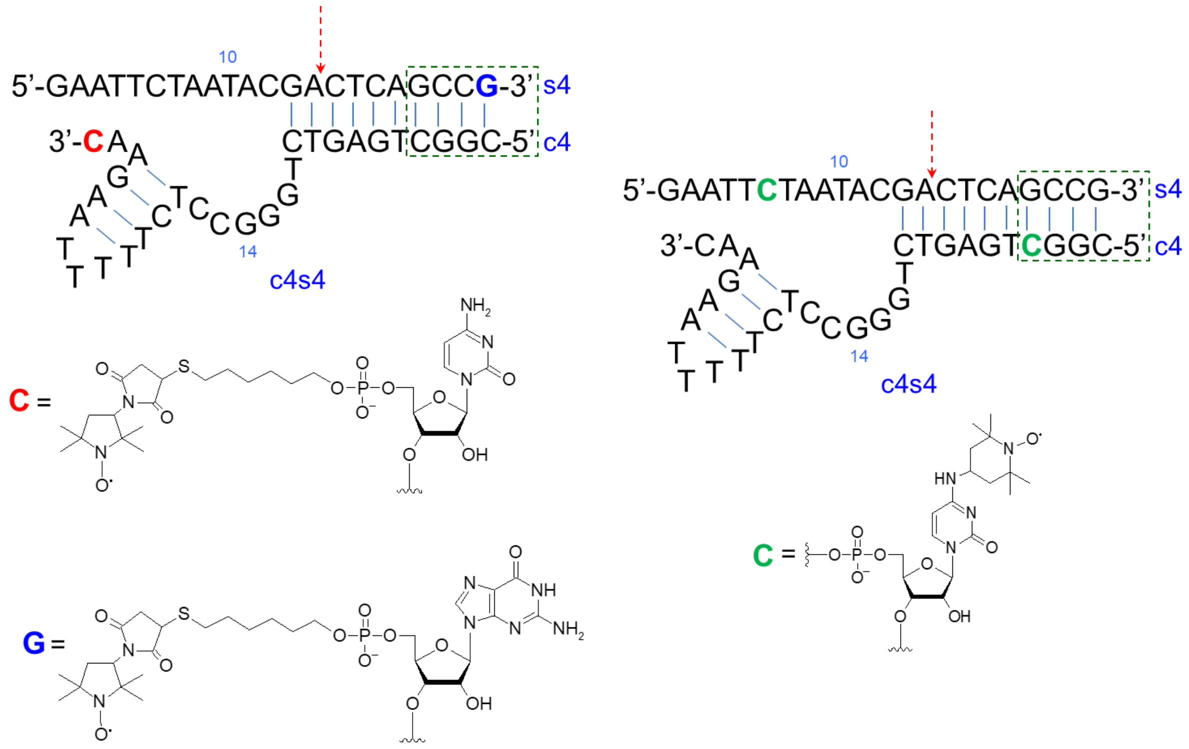

Figure S8. Spin labeled c4s4 oligomer and structures of nitroxide spin labels. On the left side the oligomers purchased from Eurogentec®, while on the right side the oligomers obtained with the procedure published elsewhere (reference 26-27 of the main text).

Supporting Information S9

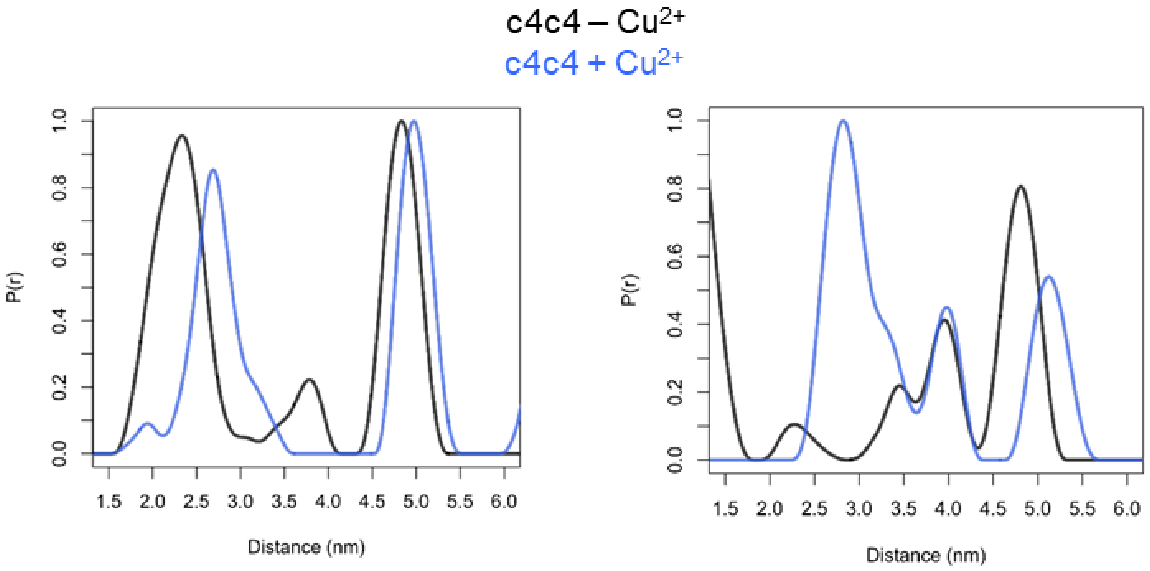

Figure S9. Distance distributions obtained by PELDOR/DEER analysis, by using spin-labelled oligos purchased from Eurogentec®, on the left side, and with the cytosine-labelled residues (right side).

Supporting Information S10

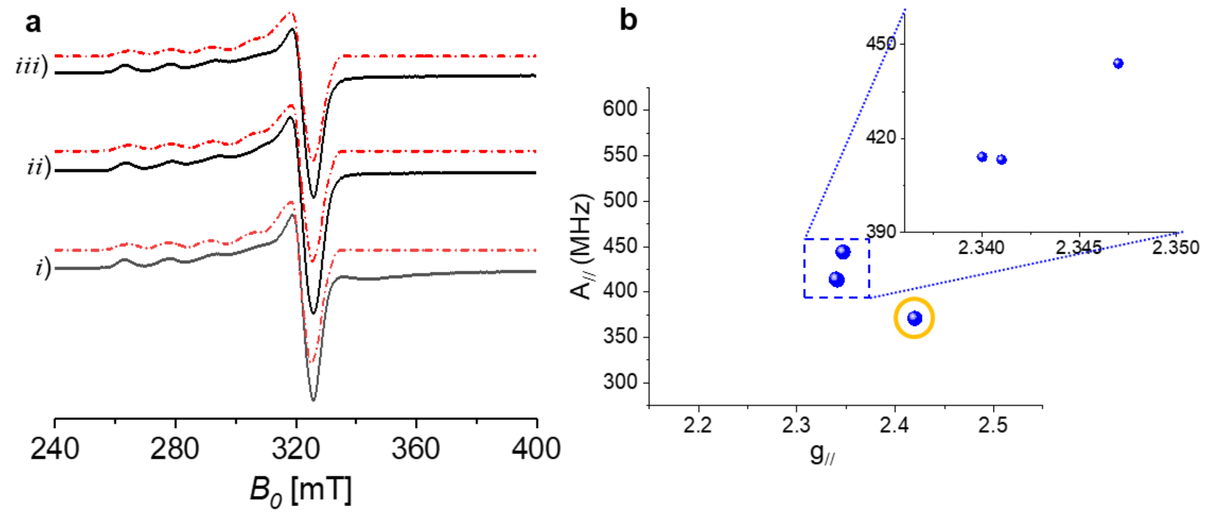

Table S1

(*iii*) c4s4-s4-GUA14-^{15}N	g (±0.005)	A (±10) (MHz)				
		Cu^{2+}	^{14}N	^{14}N	^{15}N	^{1}H
**1-x**	2.070	86	106	46	29	33
**2-y**	2.068	5	2	17	18	11
**3-z**	2.340	414	18	93	85	6

(*ii*) C4S4-c4-GUA13-^{15}N	g (±0.005)	A (±10)(MHz)				
		Cu^{2+}	^{14}N	^{14}N	^{15}N	^{1}H
**1-x**	2.071	77	113	60	34	36
**2-y**	2.067	20	20	15	11	10
**3_z**	2.347	444	30	100	100	5

(*i*) c4s4	g (±0.005)	A (±10)(MHz)				
		Cu^{2+}	^{14}N	^{14}N	^{14}N	^{1}H
**1-x**	2.07	78	100	50	30	35
**2-y**	2.067	5	10	10	15	17
**3-z**	2.341	413	28	88	100	5

Figure S10. CW-EPR spectra for Cu-c4s4 sample at X-Band, measured at 120K. The red dotted lines are the fitting of the experimental spectra (black line). (*i*) c4s4 unlabeled sample, simulated using ^{63}Cu, ^{1}H and ^{14}N, (*ii*) c4s4 isotopic labeled by ^{15}N at Guanosine residue-13 of the substrate (s4); (*iii*) c4s4 isotopic labeled by ^{15}N at Guanosine residue-14 of the catalyst (c4). **b**, The Peisach-Blumberg plot; the hyperfine coupling with ^{63}Cu for the c4s4 systems has been plotted (as function of $g_{//}$, including the references ($CuCl_2$, circled blue point). **c**, Table summarizing the hyperfine couplings with ^{63}Cu, ^{14}N, ^{1}H and ^{15}N.

Supporting Information S11

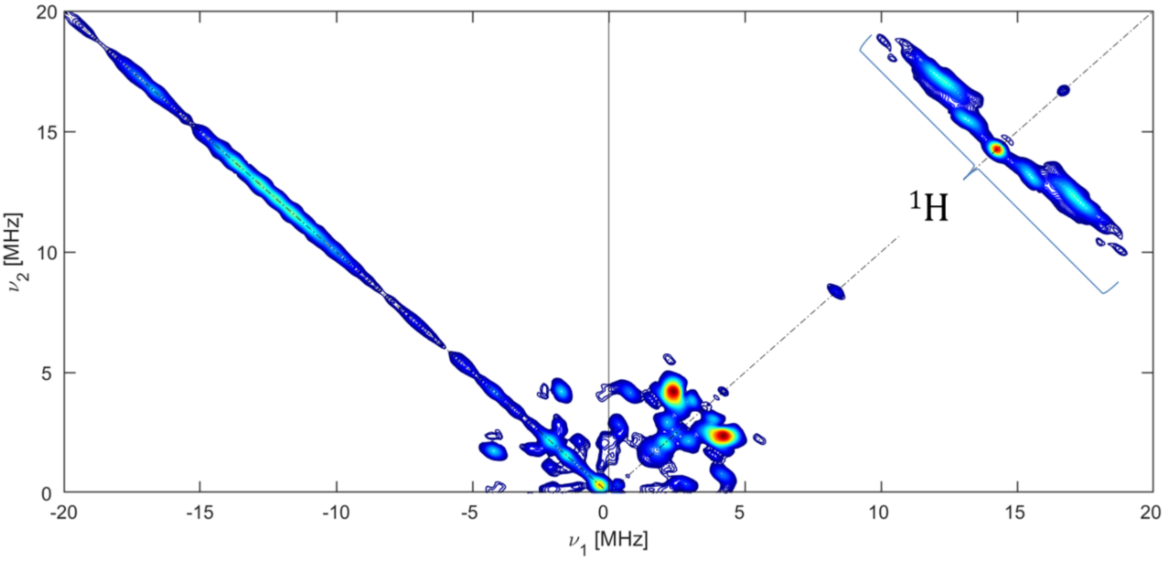

Figure S11. HYSCORE spectra recorded at X-band (9.7 GHz) for c4s4 unlabeled sample, where two main regions can be identified, for ^{14}N, and ^{1}H nuclei, respectively.

Supporting Information S12

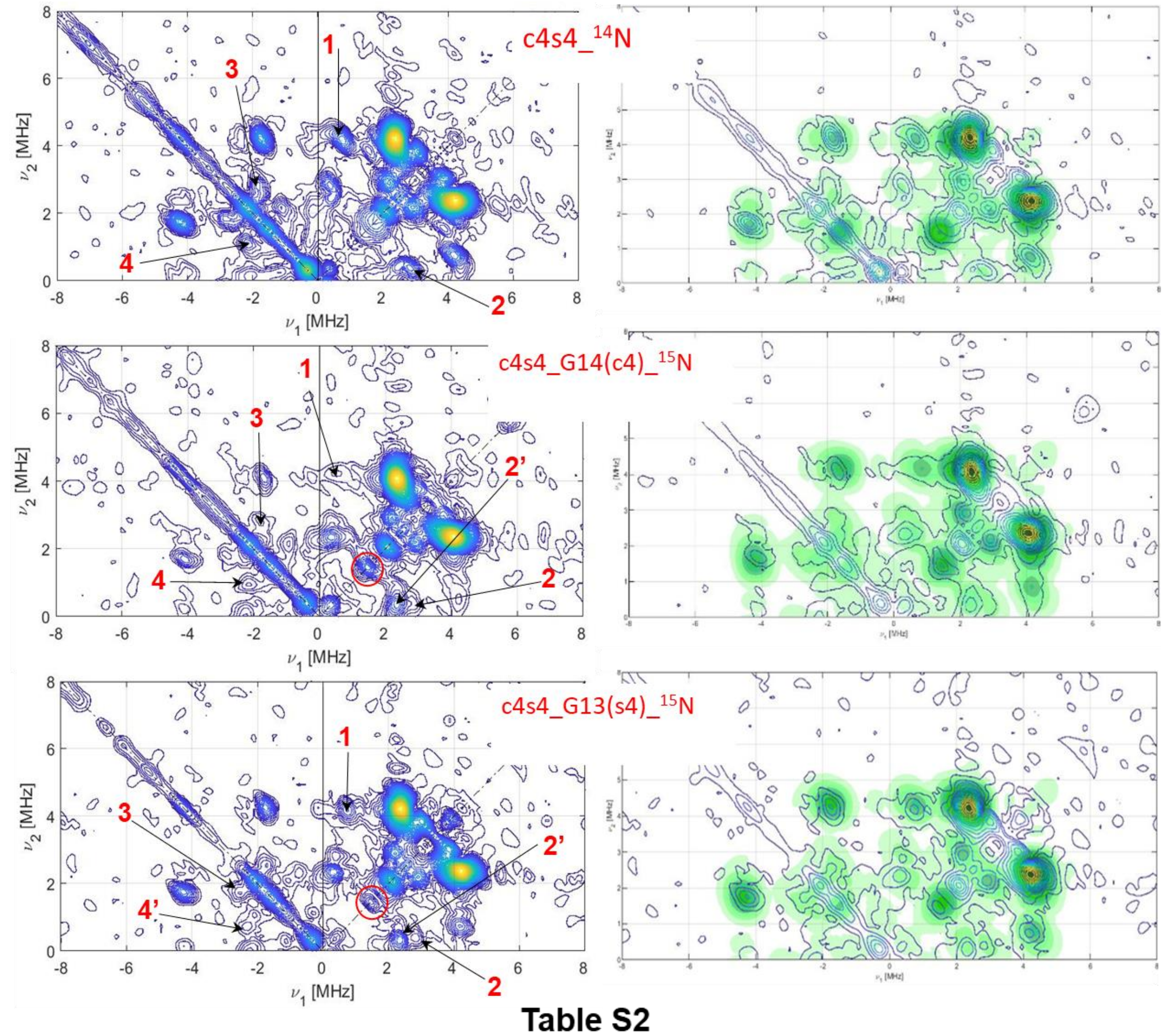

Table S2

Samples	c4s4		C4S4-c4-GUA13-^{15}N		C4S4-s4-GUA14-^{15}N	
**Parameters**	^{14}N	^{14}N	^{14}N	^{15}N	^{14}N	^{15}N
	0.98	0.98	0.98	2.02	0.98	2.14
$\boldsymbol{A}$ ($\pm$**0.07**)(**MHz**)	1.64	1.64	1.64	1.50	1.64	1.32
	2.93	2.93	2.93	1.08	2.93	1.40
	3.03	3.03	3.03	0	3.03	0
$\boldsymbol{Q}$($\pm$**0.07**)(**MHz**)	0.99	0.99	0.99	0	0.99	0
	1.00	1.00	1.00	0	1.0	0

Figure S12. (*Top*) HYSCORE spectra recorded at X-band (9.7 GHz) (*left*) and corresponding fit (*right*) for (*i*) c4s4 unlabeled sample, simulated using ^{63}Cu, ^{1}H and ^{14}N, (*ii*) c4s4 isotopic labeled by ^{15}N at Guanosine residue-13 of the substrate (s4); additional peaks with respect to the unlabeled structures are highlighted on (*i*) and (iii). (*Bottom*) **Table S12.** Main parameters used for HYSCORE fitting procedure using ^{63}Cu, ^{14}N, ^{1}H and ^{15}N.

Supporting Information S13

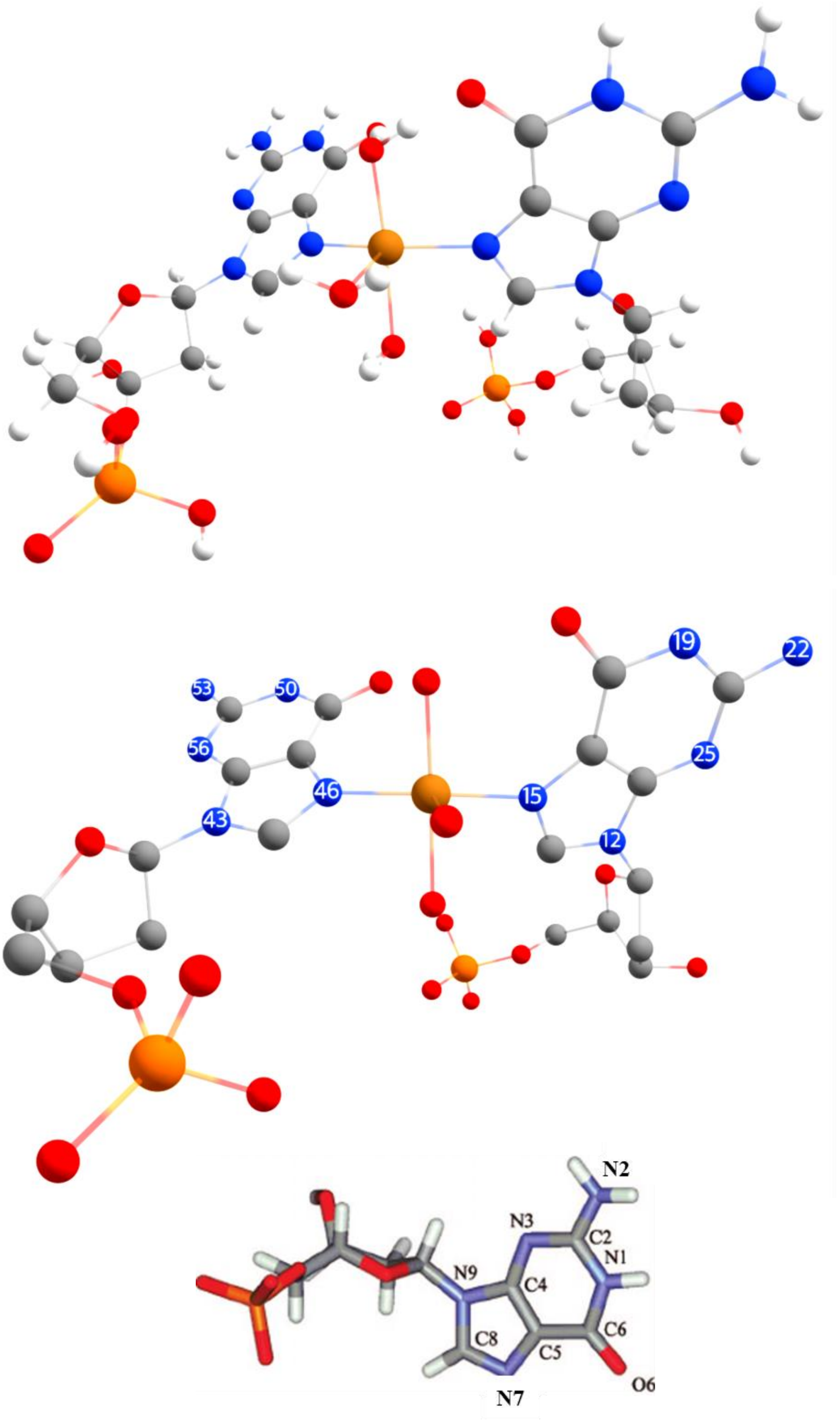

Figure S13. DFT-model for CopperG13G31 obtained from geometry optimization with ^{1}H (Top) and without ^{1}H (Bottom) removed for clarity. The numbering for the monomeric deoxy-guanosine is reported: N7 and N9 correspond to N46/N15 and N43/N12, respectively.

Supporting Information S14

Table S3. Calculated g-tensor and copper hyperfine coupling constant (individual components, MHz) of DFT-optimized model for Cu^{2+}/G13/G31

g_{min}	g_{mid}	g_{max}	g_{iso}	Δg_{min}	Δg_{mid}	Δg_{max}	Δg_{iso}
2.059	2.074	2.218	2.117	0.071	0.071	0.216	0.115
A_{min}	A_{mid}	A_{max}	A_{iso}	$\Delta A_{mid-min}$	$\Delta A_{max-mid}$	$\Delta A_{max-min}$	
52	99	540	230	47	441	488	

Table S4. Calculated nitrogen hyperfine coupling constants (individual components A, MHz) of DFT-optimized model for the complex Cu^{2+}/G13/G31 (Numbering of deoxy-guanosine is reported in Supporting Information S13).

	A_{min}	A_{mid}	A_{max}	A_{iso}
**N12**	0.89	1.15	1.70	1.24
**N15**	29.39	30.29	38.44	32.71
**N19**	0.02	-0.10	-0.13	0.07
**N22**	0.00	0.01	0.05	0.02
**N25**	0.15	-0.16	-0.21	-0.07
**N43**	0.80	1.09	1.60	1.16
**N46**	31.24	32.43	41.02	34.90
**N50**	0.02	-0.10	-0.14	-0.07
**N53**	0.00	0.00	0.04	0.01
**N56**	0.15	-0.19	-0.25	-0.10

Table S5. Cartesian coordinates of DFT-model for CopperG13G31 (Q = 2, S = 1/2).

P	63.99225109375705	72.21607116843069	33.05698285479701
O	63.04194011368811	73.34584295673139	33.18423192679151
O	65.20009153130135	72.64582145825418	32.09482546676109
C	66.28370854589195	71.72782281101317	31.77281688207431
H	65.86535731131491	70.81362766815656	31.32748288187128
H	66.83044870037101	71.46738921384269	32.69156643967920
C	67.23268250045662	72.39149679167288	30.79509265768837
H	68.09322671824502	71.71241660941678	30.66927409387164
O	66.57651549347412	72.55745237525400	29.51038974653775
C	66.79177361122989	73.89394529055447	29.04305622447183
H	67.66869423657913	73.93703876873455	28.37816206722008
N	65.65689503983630	74.27080294519011	28.20389107561899
C	64.35999344407789	74.45633594442116	28.60125537439789
H	64.04605365777373	74.38458320295410	29.63558535987672
N	63.55852969383257	74.73846016988674	27.58314847807223
C	64.37224980378311	74.73249879661637	26.44966528616408
C	64.10951330824574	74.94115912694667	25.06505658918539
O	63.01886026998918	75.17575988114652	24.49397933894525
N	65.27229351151270	74.85329448512384	24.29203254658650
H	65.13684697584722	75.00466961918148	23.29079718556064
C	66.53654097819170	74.56552421008990	24.76446855788878
N	67.54245214157524	74.52336224586320	23.87592255218630
H	67.38790170885206	74.61022294898270	22.87753937369702
H	68.46618859352201	74.26284212151288	24.20409565078922
N	66.77869422619297	74.34268547839611	26.05556565685429
C	65.69013839064397	74.44127590982970	26.82998033992891
C	67.76528172048945	73.78111476802295	31.20932454643155
H	67.57891737737907	73.98752310288866	32.27433916460354
C	66.99026201974509	74.74131856244371	30.29961794464557
H	66.02146023237819	74.98691899696004	30.75632486530512

H	67.53824035916874	75.67140954814502	30.10358782571109
P	58.35623973122456	79.90010622038449	30.86933780567100
O	57.66504077477755	81.07257660850583	31.45944051875262
O	57.53564323522832	78.52702404893633	30.86448916677105
C	56.12197120117440	78.53726451225073	30.50606338634777
H	56.01470414351411	78.85626090346739	29.45865760088062
H	55.58569851281022	79.24228571196238	31.15808785443429
C	55.54480671888884	77.14632618692214	30.68554770225599
H	54.45440940229184	77.23291168085001	30.53877815419789
O	56.07158413107234	76.25097840919153	29.66952130109430
C	56.58463083507554	75.07037454364323	30.29410330234244
H	55.84323628622019	74.25728184010937	30.26014193866468
N	57.71420955052316	74.58900572210187	29.49833199231074
C	58.88838733043190	75.25100435838299	29.25048534868054
H	59.11079218213997	76.22685067748512	29.67040003140312
N	59.68457207957476	74.56515140391357	28.44379786449987
C	58.99475913965922	73.39759393613581	28.12840792779640
C	59.30128197344029	72.30375696300553	27.26479907116131
O	60.29581724602943	72.14035997357028	26.52403773983651
N	58.29268546888532	71.33111190223327	27.29073152820574
H	58.46202025319783	70.51847760540967	26.69562906063499
C	57.11103465456023	71.40980086692824	28.00083023290257
N	56.25999580555785	70.37410989485421	27.91080930209216
H	56.41796492564858	69.59957272379555	27.27551358260569
H	55.35994628826114	70.44665563187137	28.37303618613408
N	56.80466870143664	72.45879396449494	28.76396354172022
C	57.75952671464793	73.39933053672921	28.78929271886266
C	55.81941933172766	76.47240315403775	32.05573988226884
H	56.13359073729189	77.21109182847101	32.80915646493389
C	56.92421489349152	75.47131102517316	31.73125390819246
H	57.90212460075055	75.96825079256696	31.77019316228955
H	56.92584119946598	74.61809158107280	32.41981665986214
Cu	61.56926225451176	75.03891051474133	27.88796366014224

O	61.59425157913451	77.39174433278251	27.62964840531266
H	62.29333115437100	77.66510840018174	27.00705515216016
H	60.76499073728417	77.70904103801993	27.22639067662417
O	62.04398764425795	75.70523518398720	29.80196856156244
H	61.87703423612592	76.67013569659001	29.75103450657152
H	61.49210792511613	75.36571443724125	30.53325589097865
O	61.03722400191980	74.61499258005607	26.02750112457519
H	61.81581455892334	74.80380834705977	25.38734409278170
H	60.86447436819521	73.61878962894303	26.06592700775134
O	58.78858274372906	80.03628864161081	29.33362092488280
H	59.04507567104699	80.94992409551988	29.09721268934792
O	63.41438814351164	70.82491089198199	32.51345130390355
H	62.83918284183068	70.93959995804605	31.73160697731918
O	59.73037694299394	79.45819442785604	31.56092185563576
H	59.68391822144928	79.49145766106497	32.53732442543861
O	64.63112171610125	71.63052464008373	34.40258963137151
H	64.96355832053310	72.33284554589525	34.99568677989011
O	54.68200661388551	75.73098429783020	32.52122138148435
H	53.97677569805080	76.36840943687501	32.73862902725651
O	69.17622932279919	73.78418009931255	30.93747714200575
H	69.52676821184831	74.65226004316030	31.21076640996810

Supporting Information S15

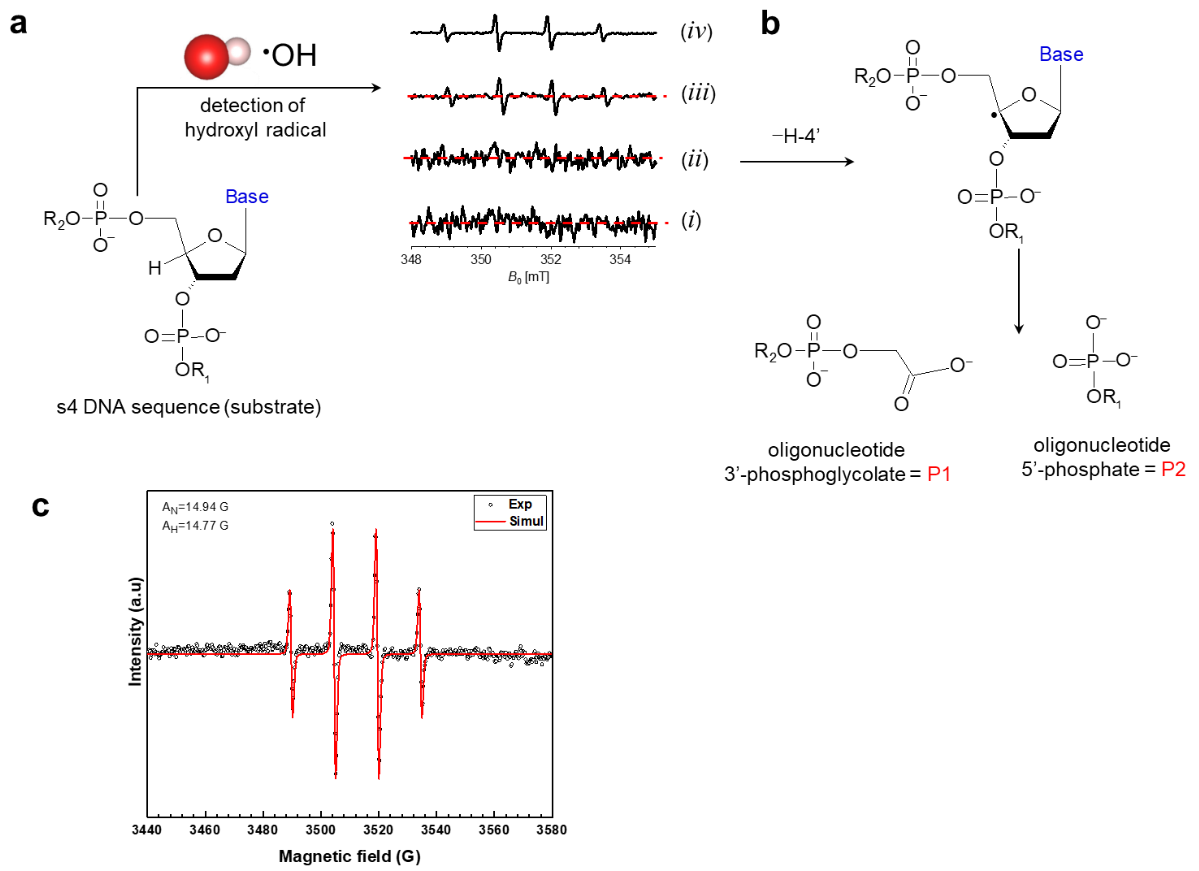

Figure S15. a) EPR spin-trap experiments on c4s4, confirming the generation on the HO• [DMPO-OH adduct]• radical species (as for a Fenton-type reaction); the spin-trap experiments carried out on the buffer solution without (*i*) and with DMPO (*ii*) do not show the production of hydroxyl radical, while in the presence of Cu^{2+} (*iii*) and Cu^{2+}/c4s4 (*iv*), the typical signal of hydroxyl radical is observed. **b**) Proton abstraction from C4' position on the ribose ring (under aerobic conditions) is here reported in una simplified scheme (selected steps are omitted). The product 3'-phosphoglycolate (P1) and the 5'-phosphate (P2) are the two DNA fragments derived by the selective cleavage. **c**) Fitting of the hydroxyl radical, with main values of hyperfine couplings reported on the inset.

Table S6.

	D(μm²/s)	*MW*(g/mol)	Log(*D*) (a.u.)	Log(*MW*) (a.u.)
**H_2O**	2.3×10^{-9}	18	– 8.64	1.26
**c_4**	55.1×10^{-12}	8897	– 10.3	3.95
**s_4**	68.1×10^{-12}	6704	– 10.2	3.83
**c_4s_4**	52.6×10^{-12}	15591	– 10.3	4.19
**His-dA**	579.3×10^{-12}	388	– 9.24	2.56
**P_1**	82.6×10^{-12}	4096	– 10.1	3.61
**P_2**	104.5×10^{-12}	2451	– 9.98	3.39
**P_3**	757.6×10^{-12}	191	– 9.12	2.28

Table S6. Diffusion coefficient and calculated molecular weights for the different fragments obtained from the cleavage reaction, including the references analyzed by DOSY (H_2O, c4, s4 and histamidine monomer).

Supporting Information S16

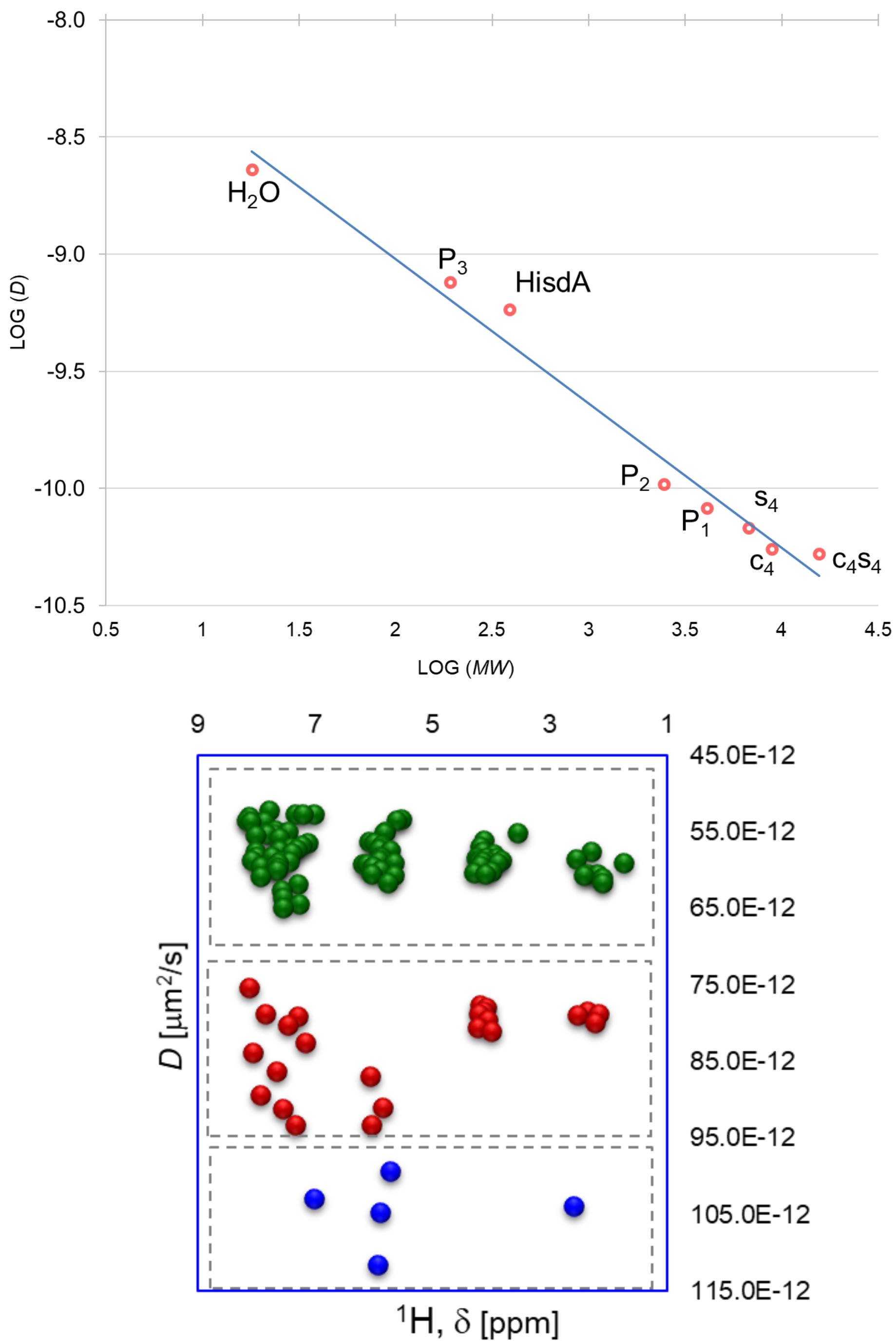

Figure S16. Log(Molecular Weight) versus Log(Diffusion coefficient) obtained by the NMR experiments (details into the table S3 / supporting information S14).

Supporting Information S17

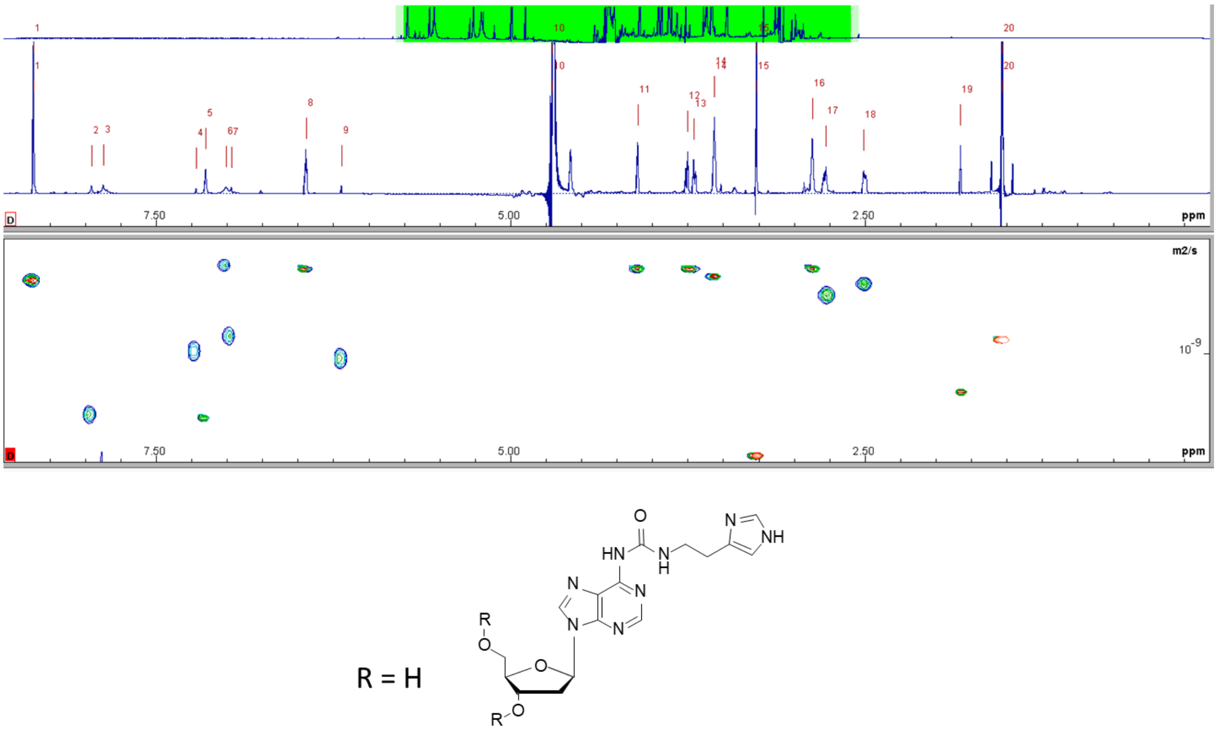

Figure S17a. NMR spectrum (900 MHz) monomeric guanosine

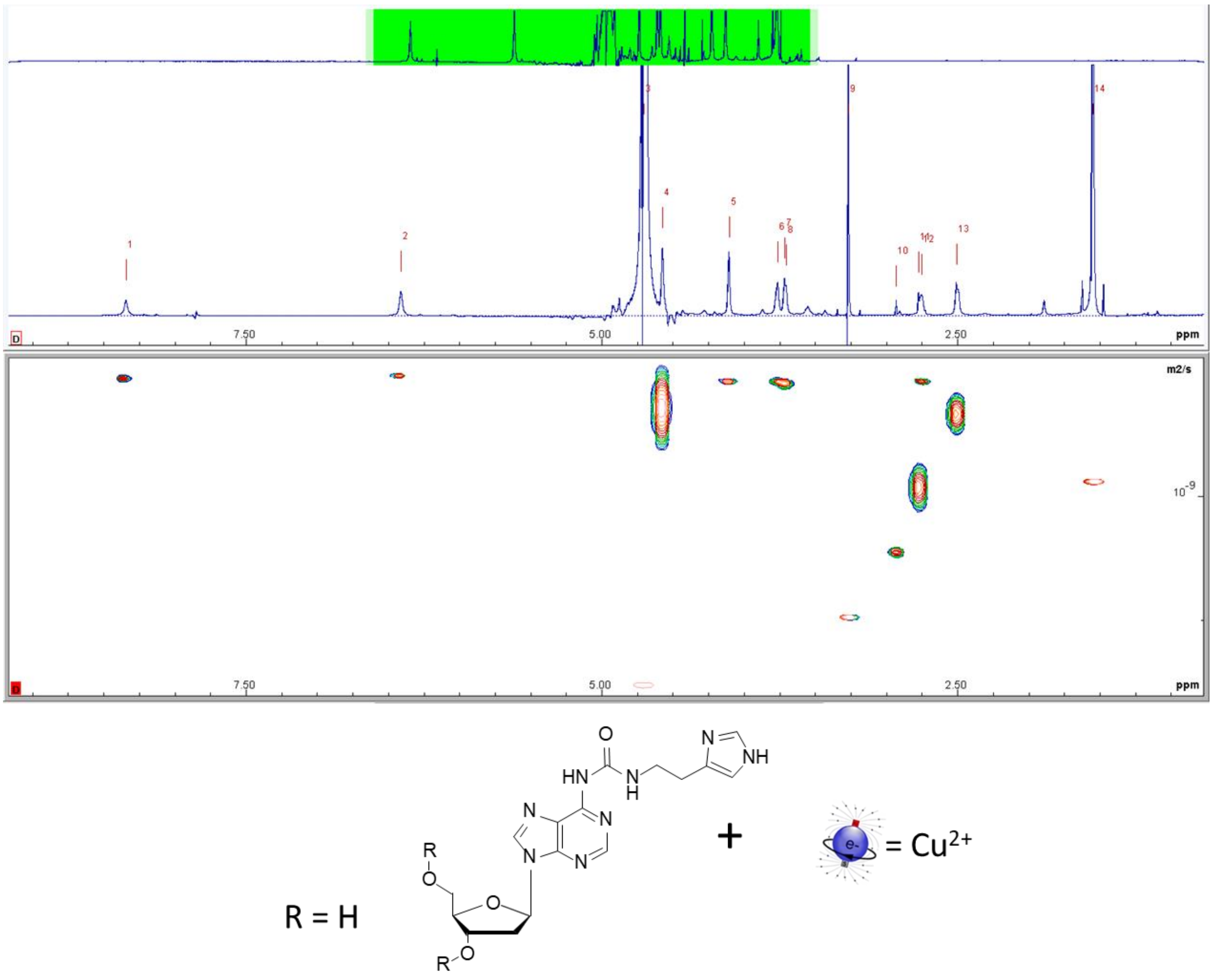

Figure S17b. NMR spectrum (900 MHz) monomeric guanosine with Cu^{2+}

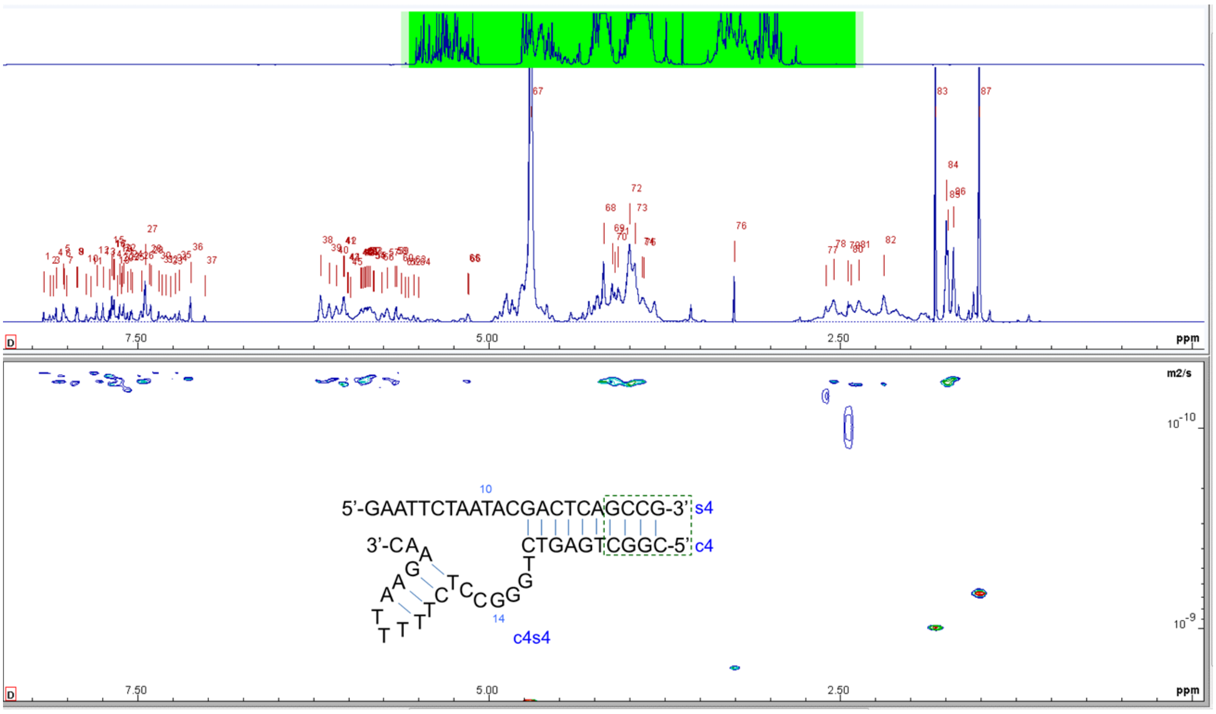

Figure S17c. NMR spectrum (900 MHz) c4s4

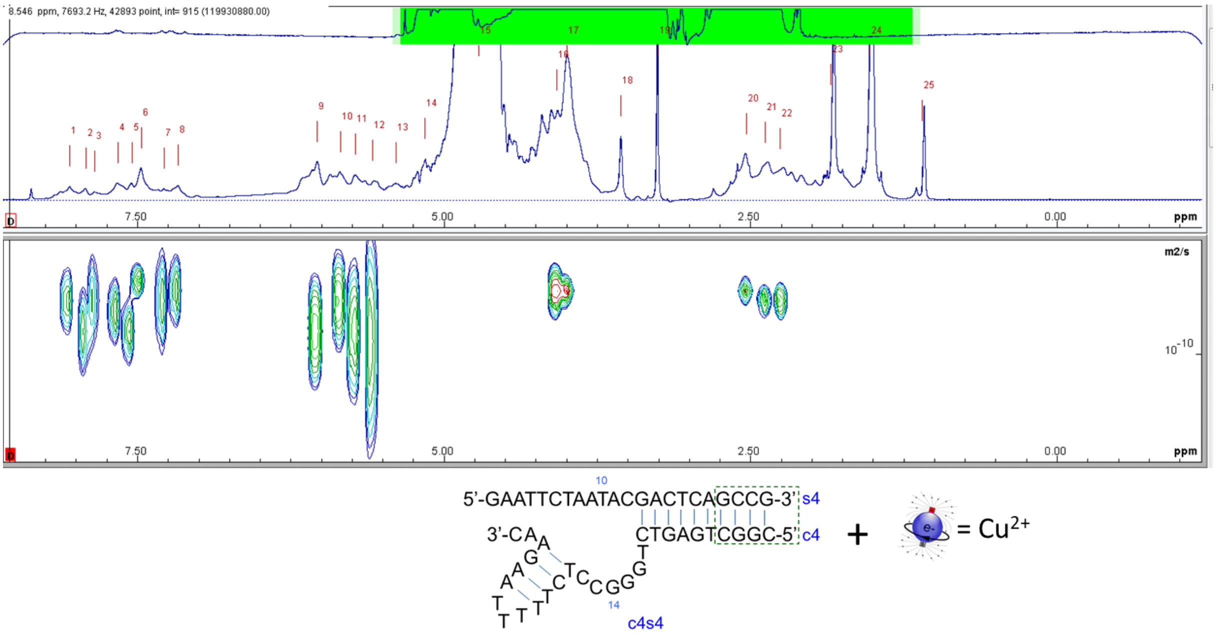

Figure S17d. NMR spectrum (900 MHz) c4s4 with Cu^{2+}

Supporting Information S18

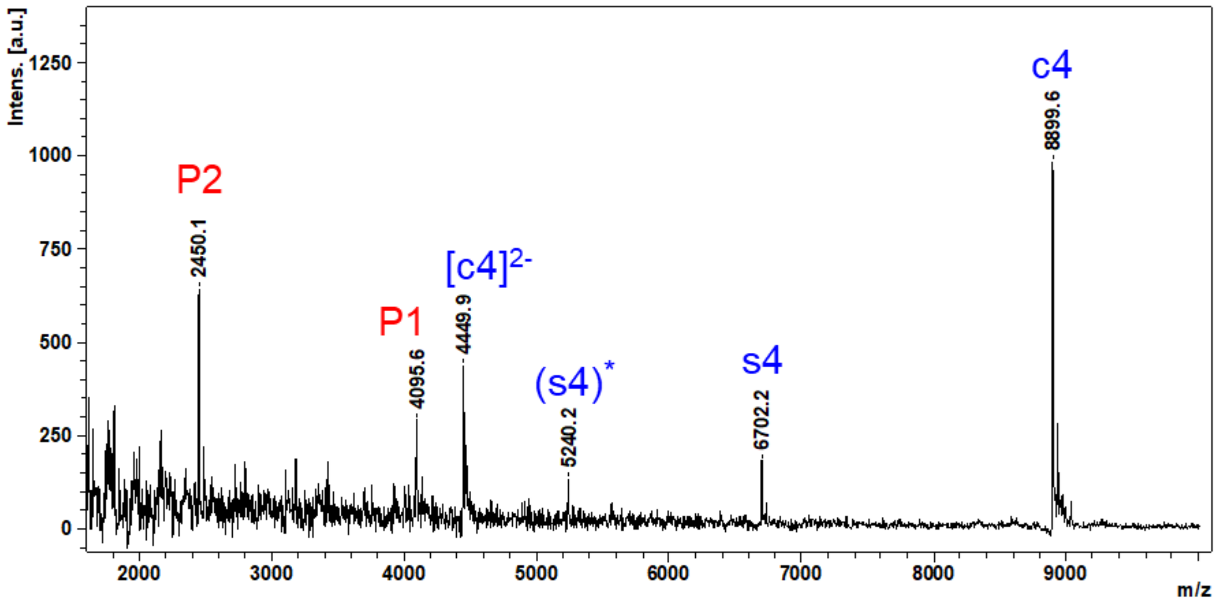

Figure S18. MALDI-TOF for the cleavage of c4s4

Supporting Information S19

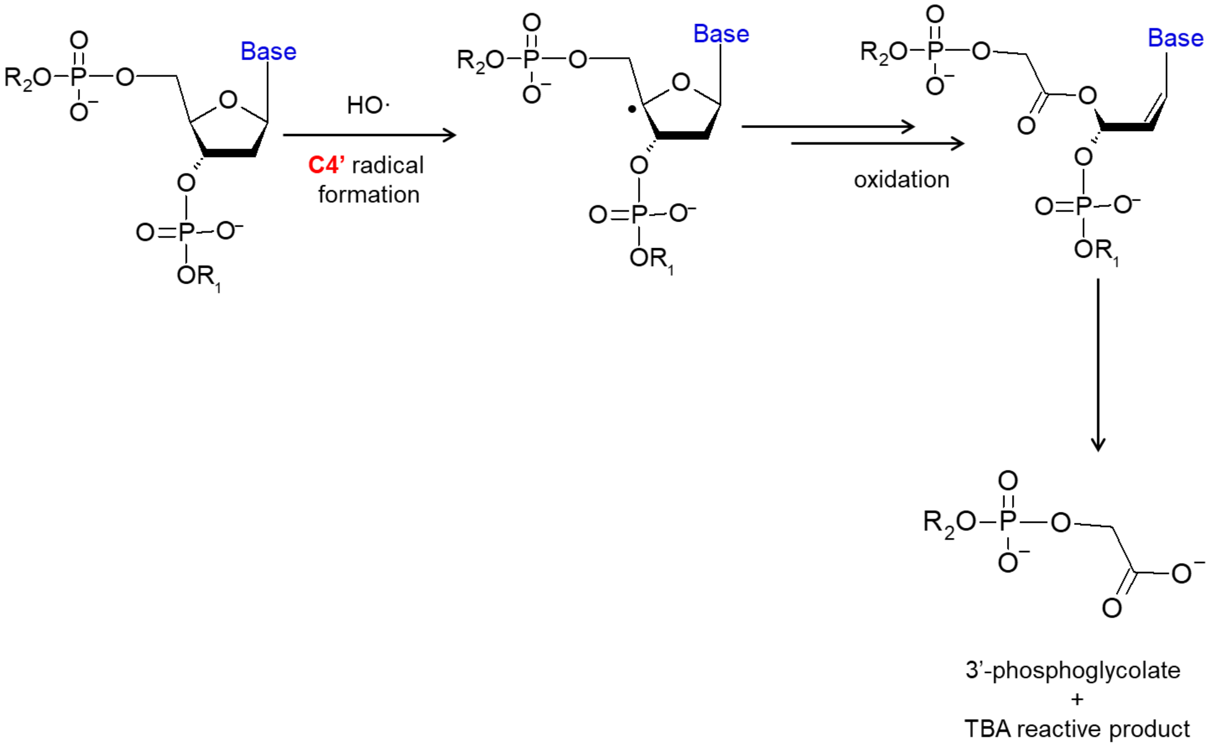

Figure S19. Hydrogen abstraction from the C4'-site

Supporting Information S20

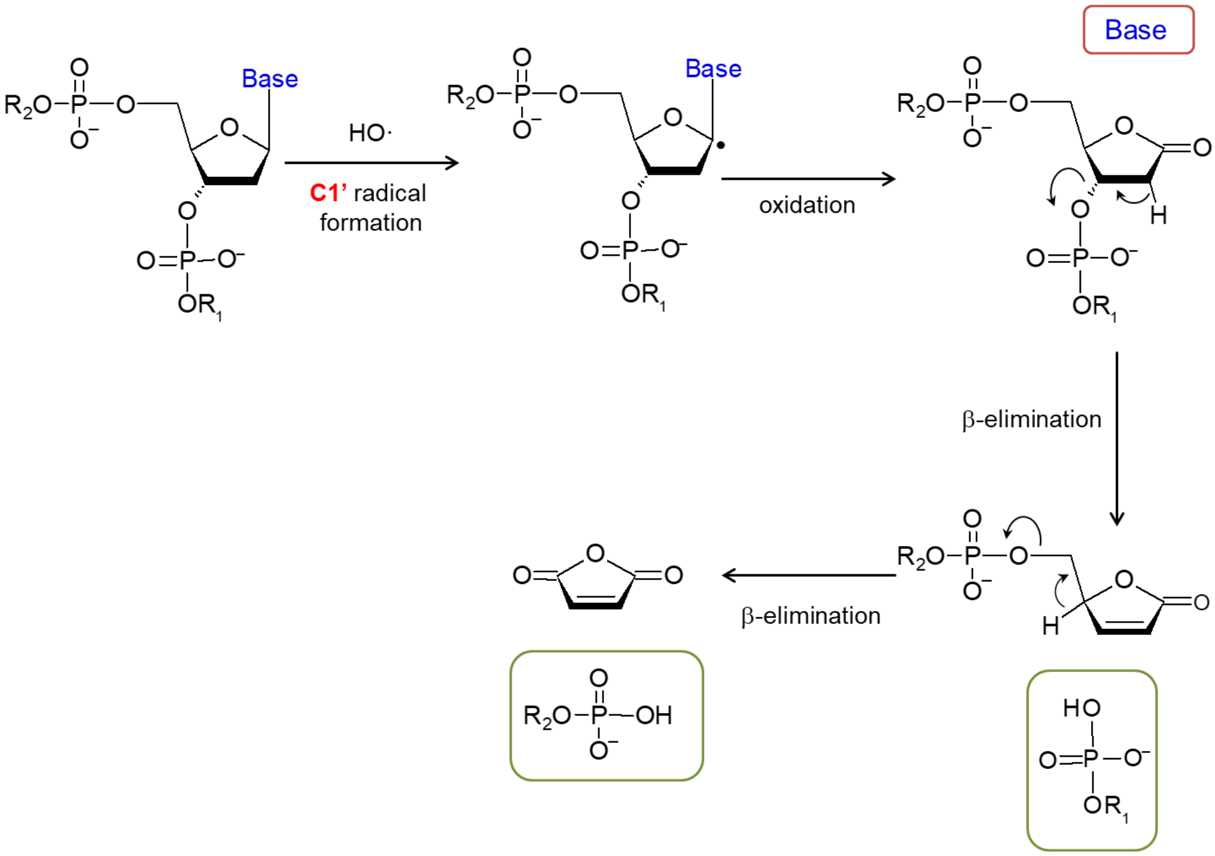

Figure S20. Hydrogen abstraction from the C1'-site

Supporting Information S21

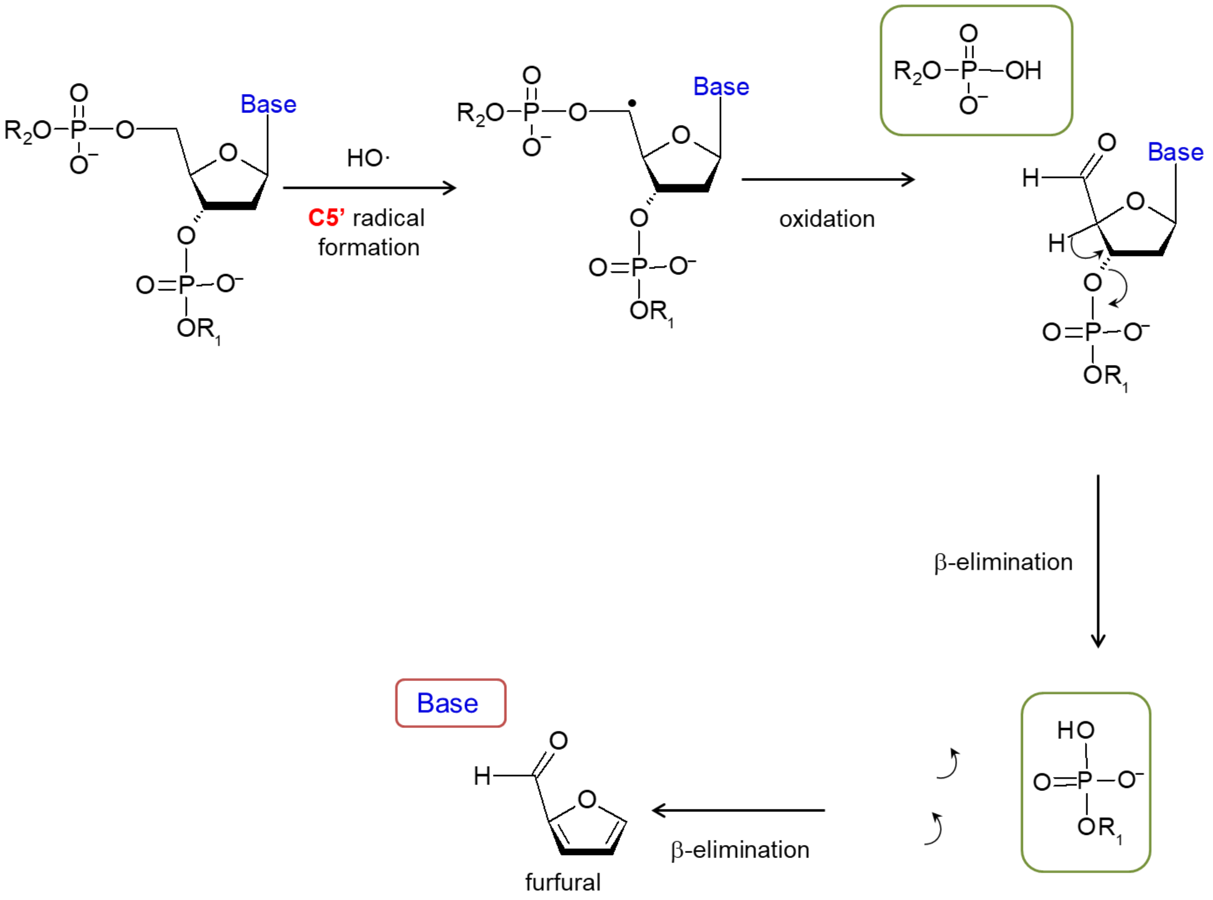

Figure S21. Hydrogen abstraction from the C5'-site

Supporting Information S22

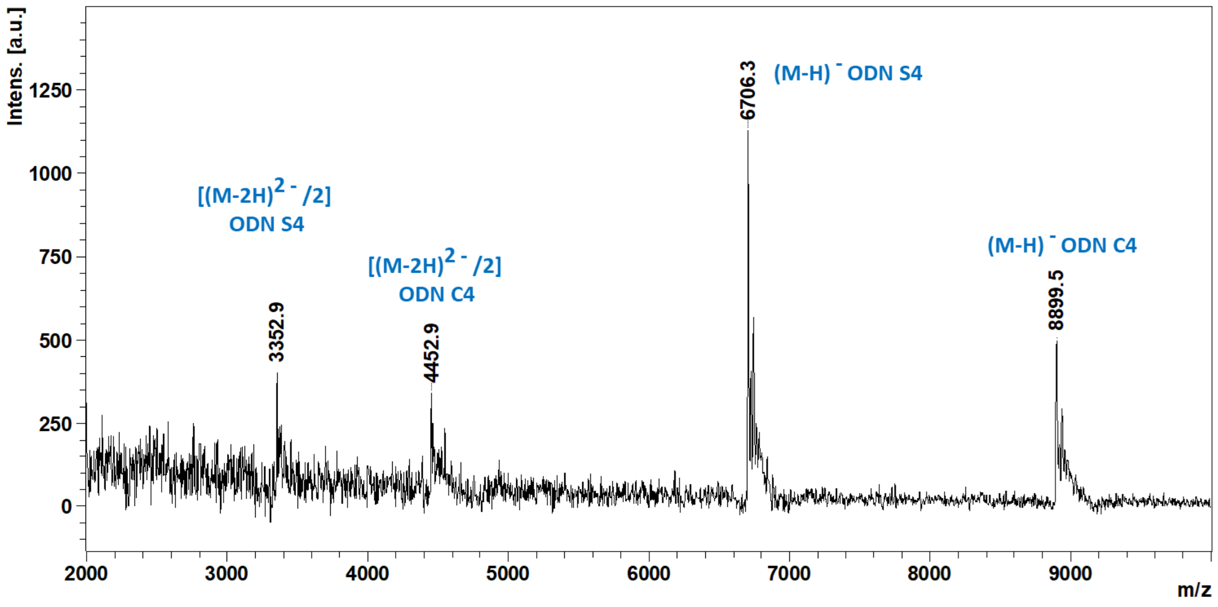

Figure S22. MALDI-TOF on the c4s4 double-strand (before cleavage)

Supporting Information S23

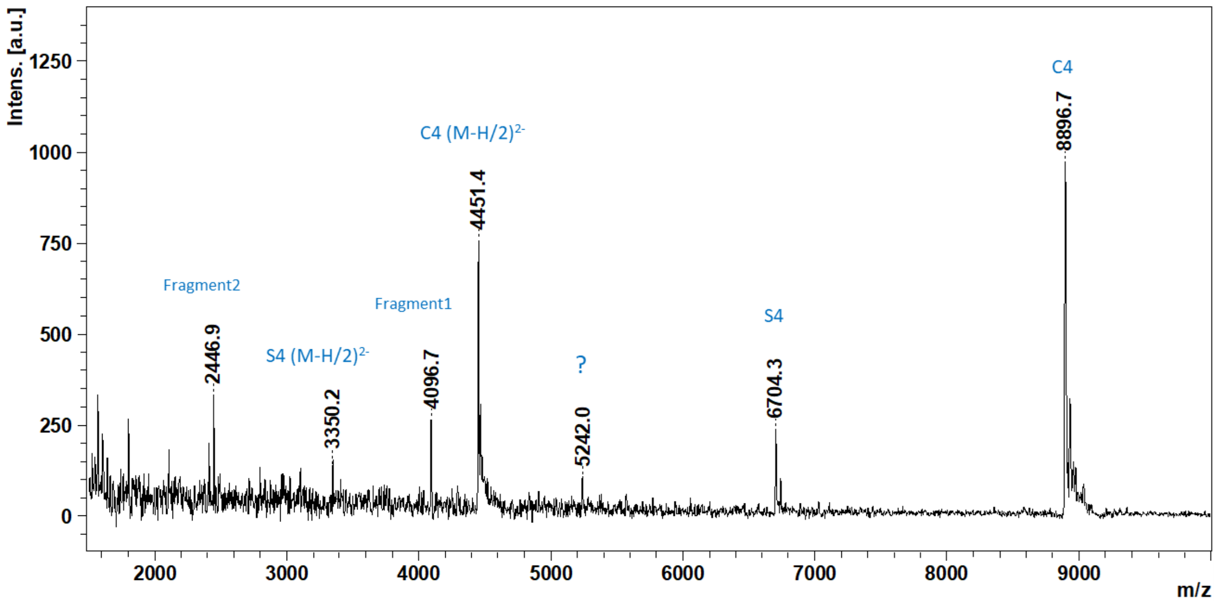

Figure S23. MALDI-TOF on the cleaved c4s4 (experiment repeated as for Supporting information S17)

Supporting Information S24

5'-GGATTCTAATACGACTCAGCCG-3' MW 6703,4

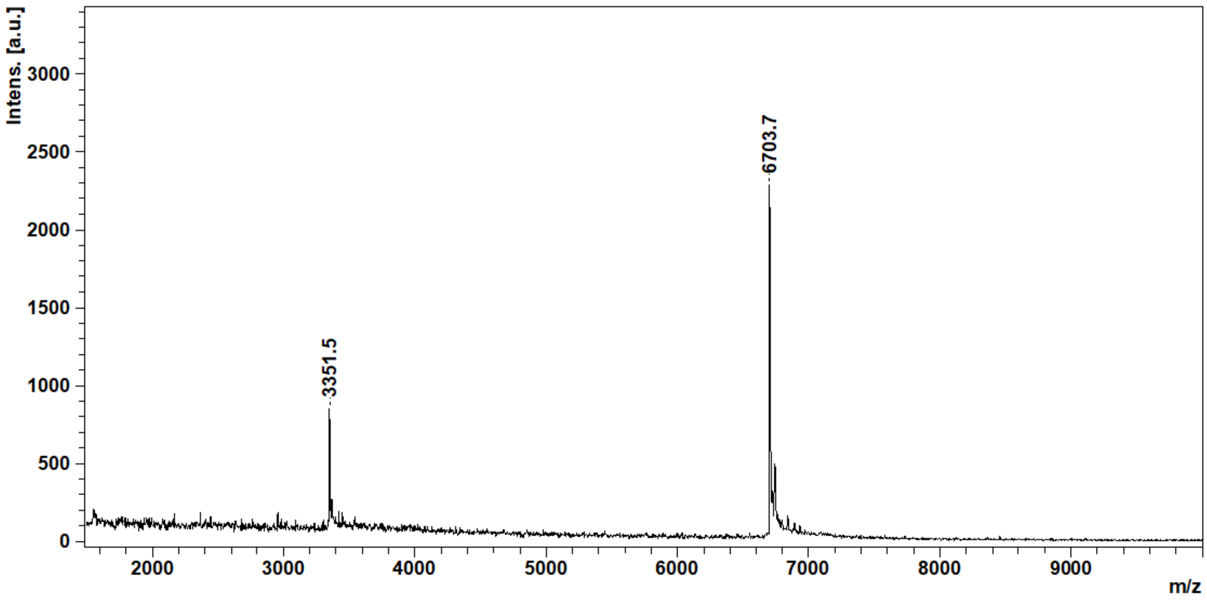

Figure S24. MALDI-TOF control experiment on s4

Supporting Information S25

5'-CGGCTGAGTCTGGGCCTCTTTTTAAGAAC-3' MW 8899,8 Da

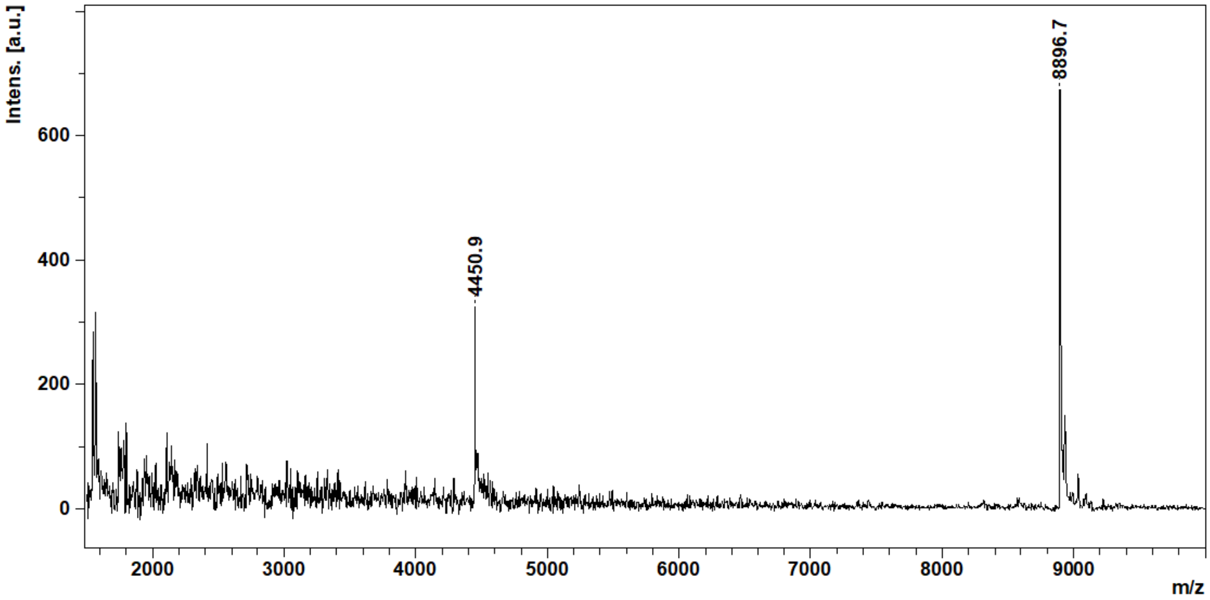

Figure S25. MALDI-TOF control experiment on c4

Supporting Information S26

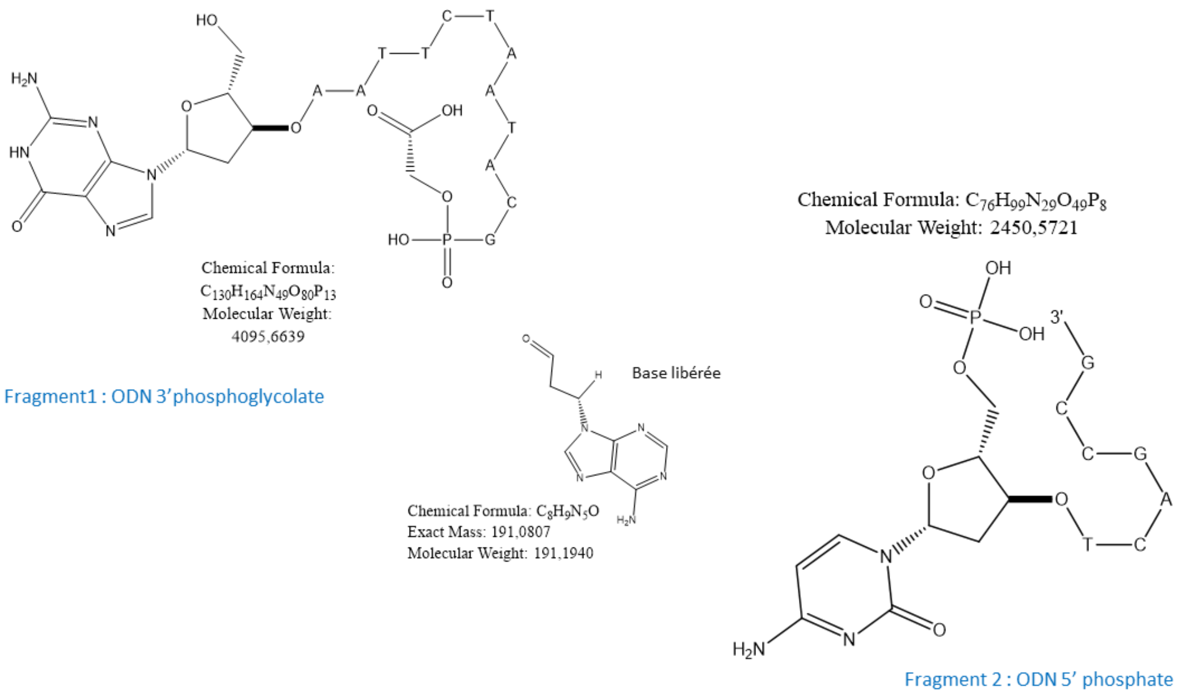

Figure S26. Products of cleavages and corresponding molecular weight

Supporting Information S27

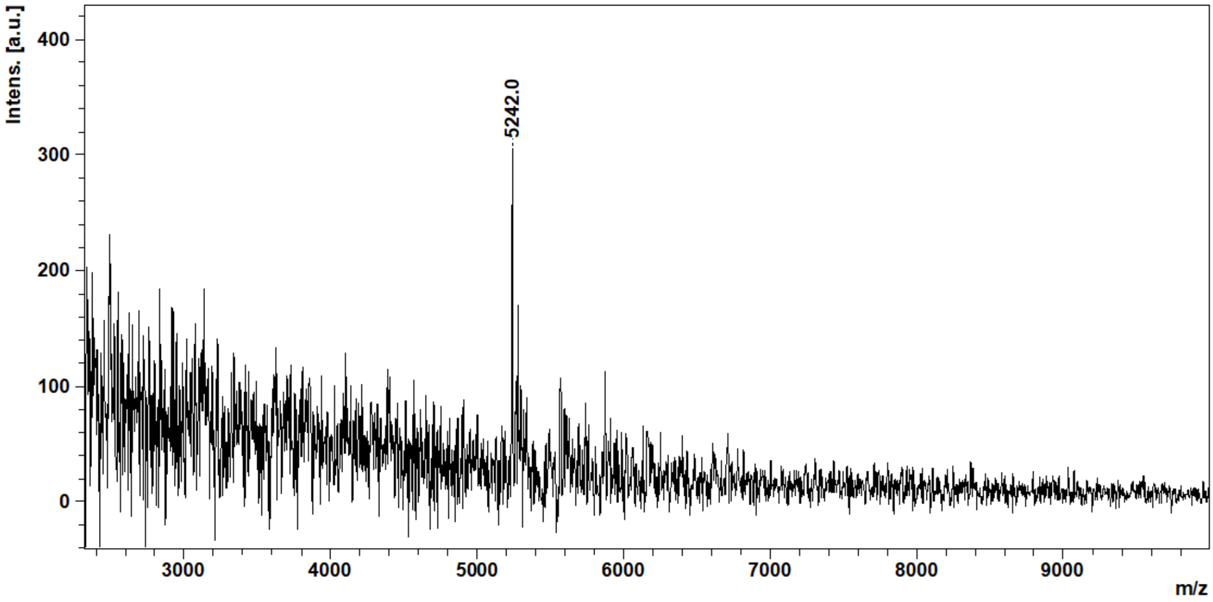

Figure S27. MALDI-TOF (zoom) on the peak 5240.8 m/z

Supporting Information S28

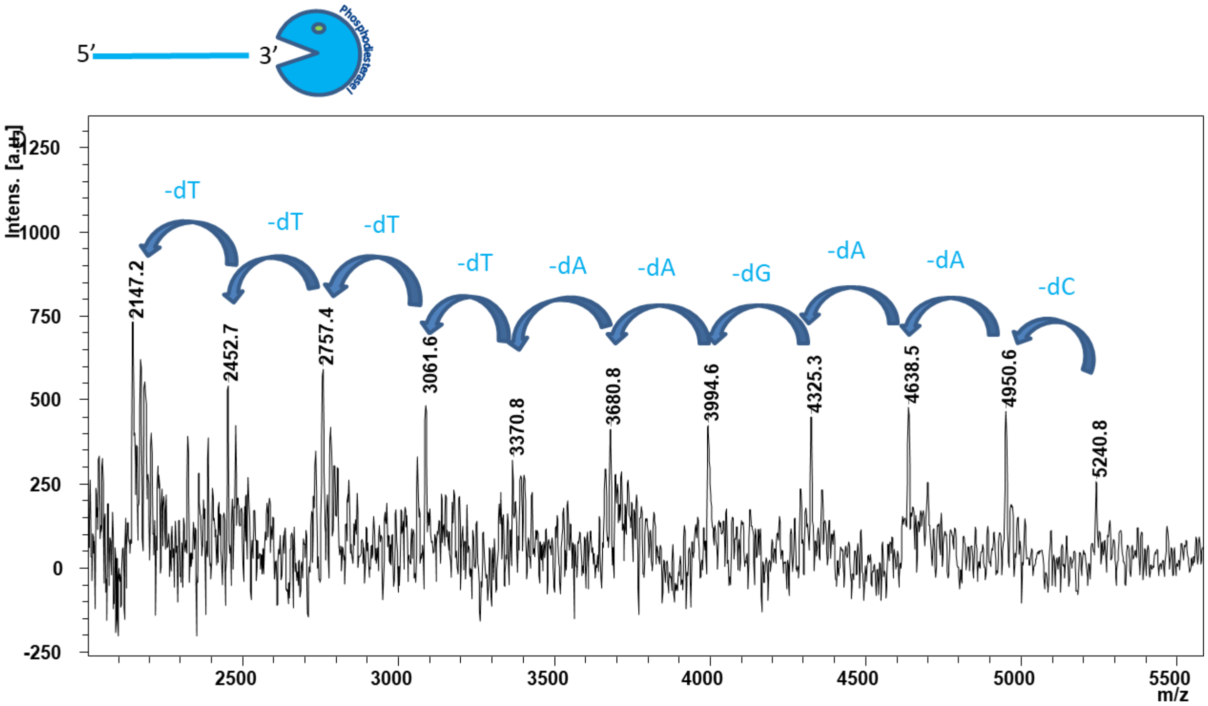

S28. Scheme of enzymatic digestion on the fragment 5240.8 m/z

Supporting Information S29

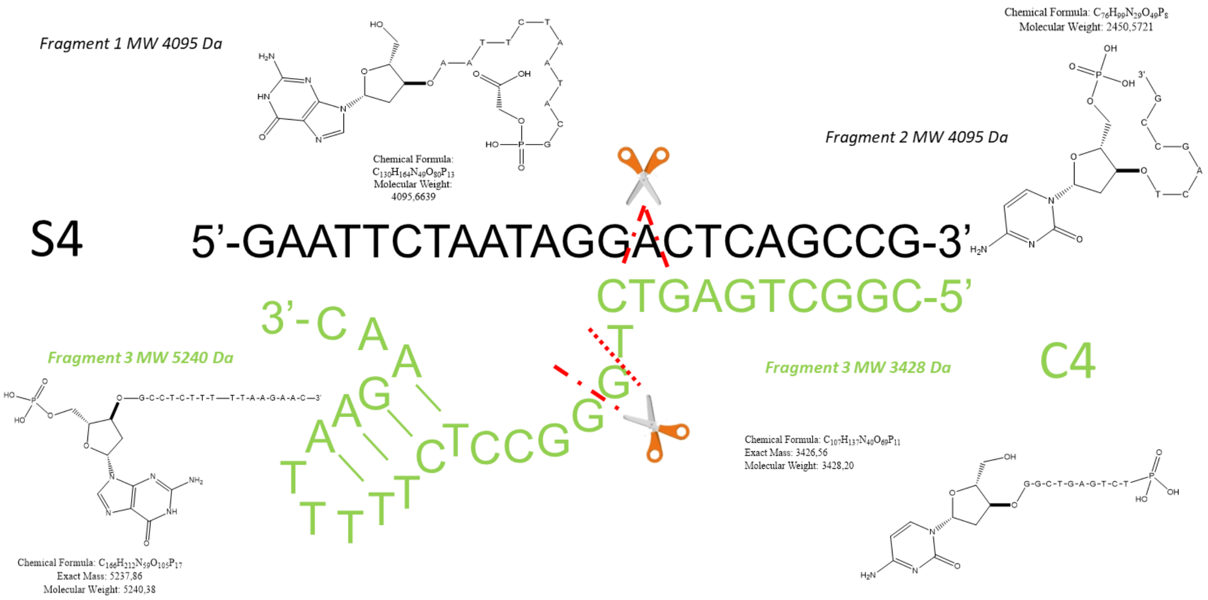

S29. Identification of the fragment 5240.8 m/z as product of catalyst decomposition

References

42 X.-J. Lu, W. K. Olson, *Nuclei Acids Research* **2003**, *31*, 5108-5121.

43 N. B. Ulyanov, W. R. Bauer, T. L. James, *Journal of Biomolecular NMR* **2002**, *22*, 265-280.

44 I. Iyani, P. S. Dans, A. Noy, A. Perez, I. Faustino, A. Hospital, J. Walther, P. Andrio, R. Goni, A. Balaceanu, G. Portella, F. Battistini, J. L. Gelpi, C. Gonzales, M. Vendruscolo, C. A. Laughton, S. A. Harris, D. A. Case, M. Orozco, *Nature Methods* **2016**, *13*, 55-58.

45 M. J. Abraham, T. Murtola, R. Schulz, S. Pall, J. C. Smith, B. Hess, E. Lindahl, *SoftwareX* **2015**, *1-2*, 19-25.

46 W. Jorgensen, J. Chandrasekhar, J. D. Madura, R. W. Impey, M. L. Klein, *Journal of Chemical Physics* **1983**, *79*, 926-935.

47 F. N. Cele, H. Kumalo, M. E. S. Soliman, *Cell Biochemistry and Biophysics* **2016**, *74*, 353-363.

48 G. Bussi, D. Donadio, M. Parrinello, *Journal of Chemical Physics* **2007**, *126*, 014101.

49 M. Parrinello, A. Rahman, *Journal of Applied Physics* **1981**, *52*, 7182-7190.

50 Daura, X, Gademan, K., Jaun, B., Seebach, D., van Gunsteren, W. F., Mark, A. E. *Angewandte Chemie International Edition* **1999**, *38*, 236-240.

51 PyMOL (RRID:SCR_000305), Molecular Graphics System, Version 2.0 Schrödinger, LLC.

52 A. Sacco, S. Heil, Self-Diffusion coefficients D of various liquids at 25°C. Courtesy (Éd.), Almanach **2007**, *80*.

53 Collectif. Diffusion Studied Using NMR Spectroscopy. E. Science, Éd (**2016**).

54 K. Haruhisa, S. Takeshi, N. Mami, S. Kayori, K. Shinichi, *Journal of magnetic Resonance* **2006**, *180*, 266-273.

55 M. Holz, H. Weingartner, *Journal of Magnetic Resonance* **1991**, *92*, 115-125.

56 M. Holz, S. Heil, A. Sacco, *Phys. Chem. Chem. Phys* **2000**, *2*, 4740-4742.

57 R. Kerssebaum, G. Salnikov, *Dosy and diffusion by NMR.* BRUKER. A tutorial for Topspin 2.0.0 Version 2.0.0 (2002-2006).

58 B. Guo, *Analytical Chemistry* **1999**, *71*, 333-337.

59 A. Capobianco, T. Caruso, A. M. D'Ursi, S. Fusco, A. Masi, M. Scrima, C. Chatgilialoglu, A. Peluso, A. *The Journal of Physical Chemistry B* **2015**, *119*, 5462-5466.

60 A. I. Taylor, V. B. Pinheiro, M. J. Smola, A. S. Morgunov, A. S. Chew, C. Cozens, K. M. Weeks, P. Herdewiin, P. Holliger, *Nature* **2015**, *18*, 427-430.

61 a) F. Neese, *Wiley Interdiscip. Rev. Comput. Mol. Sci*. **2012**, *2*, 73. b) Neese, F.; Wennmohs, F.; Becker, U.; Riplinger, *J. Chem. Phys.* **2020**, *152*, 224108.

62 a) J. P. Perdew, *Phys. Rev. B* **1986**, *33*, 8822; b) J. P. Perdew, *Phys. Rev. B* **1986**, *34*, 7406; c) A. D. Becke, *Phys. Rev. A* **1988**, *38*, 3098.

63 Weigend, F.; Ahlrichs, R. *Phys. Chem. Chem. Phys.* **2005**, *7*, 3297-3305.

64 Weigend, F. *Phys. Chem. Chem. Phys.* **2006**, *8*, 1057-1065.

65 Li, L.; Li, C.; Zhang, Z.; Alexov, E. *J. Chem. Theory Comput.* **2013**, *9*, 2126-2136

66 V. Barone, M. Cossi, *J. Phys. Chem. A* **1998**, *102*, 1995.

67 R. Gomez-Pineiro, D. A. Pantazis, M. Orio, *ChemPhysChem* **2020**, *21*, 2667.

68 D.P. Chong, ed., Recent advances in density functional methods, World Scientific, Singapore ; River Edge, N.J, 1995.

69 a) Becke, A. D. *J. Chem. Phys.* **1993**, *98*, 5648-5652. b) Perdew, J. P.; Wang, Y. *Phys. Rev. B* **1992**, *45*, 13244-13249.

70 R. J. Gómez-Piñeiro, M. Drosou, C. Decroos,1 A. J. Simaan, D.A. Pantazis, M. Orio *Inorg. Chem.* **2022**, *61*, 8022–8035.
